# Phenotype Correlations of Neurological Manifestations in Wolfram Syndrome: Predictive Modeling in a Spanish Cohort

**DOI:** 10.3390/diagnostics15243213

**Published:** 2025-12-16

**Authors:** Gema Esteban-Bueno, Luisa-María Botella, Juan Luis Fernández-Martínez

**Affiliations:** 1 UGC La Cañada (Primary Care Management Unit), Almería Periphery–Almería Health District, Andalusian Health Service (SAS), 04009 Almería, Spain; 2 Spanish Association for Research and Support to Wolfram Syndrome, 04120 Almería, Spain; 3 Centro de Investigaciones Biológicas Margarita Salas (CIB-CSIC), Consejo Superior de Investigaciones Científicas (CSIC), CIBERER U707, 28040 Madrid, Spain; 4 Group of Inverse Problems, Optimization and Machine Learning, Department of Mathematics, Oviedo University, 33007 Oviedo, Spain; jlfm@uniovi.es

**Keywords:** Wolfram syndrome, WFS1, genotype–phenotype correlation, machine learning, neurodegeneration, Random Forest, wolframin, precision medicine

## Abstract

**Background:** Wolfram syndrome (WS) is an ultrarare neuroendocrine disorder caused by pathogenic variants in WFS1, frequently leading to progressive neurological, autonomic, and cognitive impairment. Anticipating neurological trajectories remains challenging due to marked phenotypic variability and limited genotype–phenotype data. **Methods:** Forty-five genetically confirmed patients with WS were evaluated between 1998 and 2024 in Spain. All WFS1 variants were systematically classified by exon, zygosity, protein-level functional impact, and predicted wolframin production (Classes 0–3). Machine learning models (Random Forests with engineered gene–gene interaction terms) were applied to predict neurological manifestations and identify the strongest genetic determinants of symptom severity. **Results:** Neurological involvement was present in 93% of patients. The most prevalent manifestations were absence of gag reflex (67%), gait instability (64%), dysphagia (60%), and sialorrhea (60%), followed by dysmetria (56%), impaired tandem gait (53%), anosmia (44%), dysarthria (44%), and adiadochokinesia (42%). Most symptoms emerged in early adulthood (23–26 years), whereas cognitive decline occurred later (29.9 ± 12.2 years). Homozygosity for truncating variants—particularly c.409_424dup16 (Val142fsX110)—and complete loss of wolframin production (Class 0; 67–83% across symptoms) were the strongest predictors of early and severe neurological involvement. Machine learning models achieved high discrimination for ataxia, gait instability, and absent gag reflex (AUC 0.63–0.86; calibrated AUC up to 0.97), identifying Mut1_Protein_Class and Mut2_Protein_Class as dominant predictors across all phenotypes, followed by coherent secondary effects from zygosity × exon interaction terms (Prod_mgm). **Conclusions:** Integrating detailed genetic classification with machine learning methods enables accurate prediction of neurological outcomes in WS. Protein-level dysfunction and allele interaction structure are the principal drivers of neurological vulnerability. This framework enhances precision diagnosis and offers a foundation for individualized surveillance, clinical risk stratification, and future therapeutic trial design in WFS1-related disorders.

## 1. Introduction

Wolfram syndrome (WS), first described in 1938 by Wolfram and Wagener under the acronym DIDMOAD (diabetes insipidus, diabetes mellitus, optic atrophy, and deafness), is a rare, progressive disease with a prevalence estimated between 1 in 55,000 and 1 in 770,000 live births, depending on population genetics and consanguinity rates [1,2]. WS is inherited primarily in an autosomal recessive pattern, although autosomal dominant and sporadic cases have also been reported [3]. Most cases (WS type 1) result from biallelic pathogenic variants in *WFS1* on chromosome 4p16.1, which encodes wolframin, an 890-amino-acid endoplasmic reticulum (ER) transmembrane glycoprotein involved in ER homeostasis, calcium signaling, redox balance, and apoptosis [4,5]. Loss-of-function variants—such as nonsense, frameshift, and large deletions—lead to absent or dysfunctional wolframin, triggering chronic ER stress, β-cell failure, and progressive neurodegeneration [5]. More than 200 different *WFS1* mutations have been reported, most clustering in exon 8, which encodes the transmembrane and C-terminal domains [3]. Truncating mutations, particularly in exons 4 and 8, have been associated with earlier onset of diabetes and optic atrophy, as well as more severe neurological manifestations [6]. A rarer subtype, Wolfram syndrome type 2 (WS2), results from biallelic variants in CISD2 on chromosome 4q22–q24. WS2 shares overlapping features with WS1 but typically lacks diabetes insipidus and may present gastrointestinal ulceration, bleeding tendency, and sensorimotor neuropathy [2]. These molecular distinctions broaden the phenotypic spectrum of WS and highlight the role of endoplasmic reticulum stress and mitochondrial dysfunction as shared pathogenic pathways.

Clinically, WS typically appears in early childhood with insulin-dependent diabetes mellitus before age 6, followed by optic atrophy around age 11 [1]. Sensorineural hearing loss usually develops during adolescence, and diabetes insipidus in the second decade of life. Neurological manifestations—including cerebellar ataxia, gait instability, dysarthria, dysphagia, dysmetria, peripheral neuropathy, anosmia, psychiatric disturbances, and progressive cognitive decline—often emerge in the third to fourth decade and are major determinants of morbidity and mortality [2,7]. Brainstem dysfunction, such as absence of gag reflex, sialorrhea, and abnormal respiratory patterns, contributes significantly to aspiration pneumonia and premature death [1].

Neuroimaging findings have reinforced WS as a primary neurodegenerative disorder. MRI volumetric studies show early involvement of the brainstem and cerebellum, with marked atrophy of the pons, medulla, and cerebellar hemispheres even in pediatric patients [8,9]. Diffusion tensor imaging (DTI) reveals widespread microstructural abnormalities in corticospinal tracts, cerebellar peduncles, and optic radiations, suggesting early axonal degeneration [10]. Longitudinal MRI studies confirm that brainstem and cerebellar atrophy progress over time and correlate with gait instability, dysarthria, and ataxia [9]. Later cortical and hippocampal involvement have been reported, correlating with cognitive and psychiatric symptoms [8]. Strong genotype–phenotype correlations have been identified. Homozygosity for truncating variants, particularly c.409_424dup16 (Val142fsX110), is overrepresented among patients with severe neurological phenotypes, including early gait instability, absence of gag reflex, and cognitive decline [6]. Frameshift and nonsense variants producing a “wolframin class 0” phenotype (complete loss of protein) is linked to earlier onset of diabetes, optic atrophy, and more rapid neurological progression compared to missense variants that may retain partial protein activity [3]. Despite these insights, longitudinal data quantifying the prevalence, age of onset, and genetic predictors of specific neurological manifestations remain limited. Furthermore, few studies have integrated machine learning or predictive models based on functional classification of wolframin to estimate individual risk and disease trajectories.

The objective of this study is to provide a comprehensive characterization of the genetic and neurological landscape of Wolfram syndrome (WS) in a large, genetically confirmed Spanish cohort ( n = 45). We systematically classify all WFS1 variants according to their predicted protein-level consequences, exon-level mutation structure, and zygosity configuration, with the aim of establishing a biologically informed framework for interpreting allelic heterogeneity. Building on this genetic structure, we investigate the association between specific mutation classes and the breadth of neurological manifestations observed in WS—including brainstem reflex abnormalities, cerebellar motor deficits, gait and coordination problems, sensory disturbances, and cognitive involvement—to delineate patterns of selective neural vulnerability.

In parallel, we apply machine learning methods to model complex genotype–phenotype interactions that may not be captured through conventional statistical approaches. These models allow us to identify the most informative genetic predictors of neurological dysfunction, uncover hierarchical relationships among mutation features, and explore the temporal clustering of symptoms across the disease course. By integrating molecular genetics with quantitative phenotyping and advanced computational modeling, our goal is to improve risk stratification, enhance early recognition of high-severity neurological trajectories, and ultimately inform clinical monitoring and intervention strategies—particularly for complications such as dysphagia-related aspiration, which remain major contributors to morbidity and mortality in WS.

Complementary analyses from the same Spanish cohort have also documented both the prevalence and longitudinal progression of gonadal dysfunction [11], as well as the pattern of neurosensory impairment—particularly the auditory decline characteristic of Wolfram syndrome [12].

## 2. Materials and Methods

### 2.1. Study Design and Participants

The present study has been approved by the Ethics Committee of Torrecárdenas University Hospital (protocol code 75/2020; approval date 27 February 2020), and all patients signed informed consent prior to/before participation in the study.

The database was structured into three main components: (i) epidemiological variables, (ii) monitored genetic attributes and their potential interactions, and (iii) symptom class vectors. Clinical and genetic data were collected as part of a longitudinal follow-up initiated in 1998 under the supervision of the principal investigator, who also has coordinated the research team. During the early years, information was obtained from medical reports and clinical documentation provided by hospitals across Spain, directly reviewed and clinically assessed by the principal investigator. Since September 2010, patients have been directly evaluated by a multidisciplinary team specialized in Wolfram syndrome, integrated within the Almería Health District, with coordinated activity across several facilities of the Andalusian public health system, including Hospital La Inmaculada (Huércal-Overa) and, more recently, Hospital Universitario Torrecárdenas. The same principal investigator has continued the systematic collection, evaluation, and updating of the database, ensuring methodological consistency and data reliability throughout the study period.

**Figure 1:**
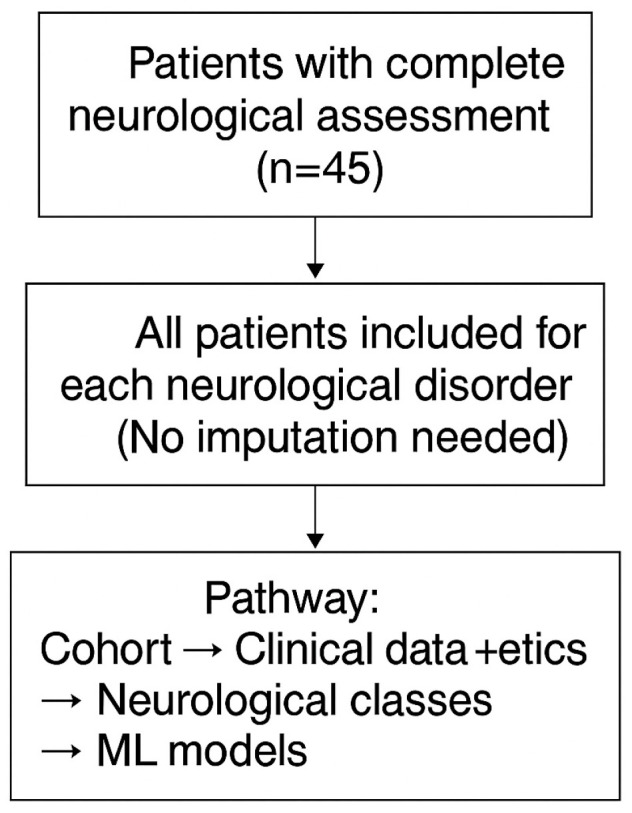
Study flow diagram. This diagram summarizes the analytic pathway for the neurological assessment dataset. All 45 patients completed a full neurological evaluation and were included in the analysis for each neurological disorder, with no imputation required. The modeling pipeline proceeded from the full cohort through clinical and genetic data integration, followed by classification into neurological groups and development of outcome-specific machine learning models.

Figure 1 summarizes the flow diagram for this study.

### 2.2. Clinical and Cognitive Assessment

Cognitive function was assessed using age-adjusted Wechsler scales (WAIS/WISC). Neurological examinations systematically evaluated brainstem, cerebellar, cortical, and peripheral domains. Each clinical manifestation—such as gait instability, dysphagia, dysmetria, or anosmia—was encoded as a binary variable (present/absent) to facilitate computational modeling.

### 2.3. The Genetic Attributes

A detailed characterization of the genetic background of the cohort is essential for accurately interpreting the relationship between WFS1 mutations and the neurological manifestations observed in Wolfram syndrome. However, comprehensive genetic information is not uniformly available for all patients, which poses inherent challenges for both modeling and clinical interpretation. Table 1 summarizes the overall genetic status of the Spanish cohort analyzed in this study.

Wolframin, encoded by the WFS1 gene, is a transmembrane protein located in the endoplasmic reticulum (ER), where it plays a fundamental role in maintaining ER homeostasis, regulating intracellular calcium levels, and protecting neurons and pancreatic β-cells from stress-induced apoptosis. Loss or dysfunction of wolframin leads to diabetes mellitus, progressive neurodegeneration, and sensory deficits including visual and auditory impairment.

To capture the functional consequences of WFS1 variants, patients were classified into four wolframin classes according to predicted protein production and folding integrity:

• Type 0: No wolframin production (both alleles carry nonsense mutations, deletions, duplications, or splice defects leading to premature termination)
• Type 1: Partial production (~50%), typically in compound heterozygotes with one missense and one truncating variant; the residual protein is largely misfolded.
• Type 2: Two missense variants resulting in misfolded proteins with partial or absent function.
• Type 3: Autosomal dominant heterozygous condition producing ~50% normal protein and ~50% misfolded protein.

This level of granularity was established given the central role of wolframin dysfunction in Wolfram syndrome pathophysiology. In our cohort, 48.9% of individuals were heterozygous carriers (Table 1).

Table 2 and Table 3 list all WFS1 variants identified at the cDNA and protein levels, respectively. These variants were subsequently incorporated into the predictive model to explore genotype–phenotype correlations.

For analytical purposes, each patient’s WFS1 variants were classified according to exon location and predicted functional impact. Specifically:

• Type_mut1_exon and Type_mut2_exon describe the type of mutation on each allele and the exon in which it occurs.
• Mut12_Exon_Class captures whether both mutations occur in exon 4, both in exon 8, or one in each.
• Mut1_Protein_Class and Mut2_Protein_Class represent the predicted protein-level effect of each allele.
• Genetic_Condition indicates whether the patient is homozygous, compound heterozygous, or triple heterozygous.
• Wolframin_Class groups patients into the four predicted protein-production classes described above (Types 0–3).

To explore potential epistatic or synergistic effects, interaction terms combining wolframin class, genetic condition, and exon-related mutation structure were generated. These include:

• Prod_wm1 = Wolframin_Class × Type_mut1_exon.
• Prod_wm2 = Wolframin_Class × Type_mut2_exon.
• Prod_wm12 = Wolframin_Class × Mut12_Exon_Class.
• Prod_wmg = Wolframin_Class × Genetic_Condition.
• Prod_mgm1 = Genetic_Condition × Type_mut1_exon.
• Prod_mgm2 = Genetic_Condition × Type_mut2_exon.
• Prod_mgm12 = Genetic_Condition × Mut12_Exon_Class.

All genetic variables and interaction terms incorporated into the machine learning model are summarized in Table 4. Throughout the manuscript, we refer to these variables using the same nomenclature defined in the model, and their meaning is explicitly detailed in this table.

### 2.4. Machine Learning Model

To investigate the predictive value of genetic features for neurological manifestations in Wolfram syndrome, we implemented a supervised machine learning pipeline based on a Balanced Random Forest (BRF) classifier [13,14] using scikit-learn’s RandomForestClassifier.

The model was configured with 500 trees (n_estimators = 500), Gini impurity as the splitting criterion (criterion = ‘gini’), unrestricted tree depth (max_depth = None), min_samples_split = 2, min_samples_leaf = 1, square-root feature subsampling (max_features = ‘sqrt’), bootstrap sampling (bootstrap = True), class weighting to account for class imbalance (class_weight = ‘balanced’), and a fixed random seed (random_state = 42). All remaining hyperparameters were left at their default scikit-learn values, and no additional hyperparameter tuning was performed. We are aware that these parameters should be systematically optimized if the model were to be deployed in a production setting; however, given the limited sample size and the exploratory nature of this work, we prioritized a simple and transparent configuration over production-level optimization.

The BRF algorithm was selected to address the inherent class imbalance present in rare-disease datasets, in which several neurological symptoms occur at relatively low frequencies. All analyses were performed in Python 3.9 using the scikit-learn [15] and imbalanced-learn libraries [16]. The feature set included all primary genetic variables, as well as all biologically defined pairwise interaction terms. This feature design enabled the model to capture both linear and non-linear genotype–phenotype relationships, including potential epistatic or synergistic effects that cannot be detected through classical regression approaches. Categorical genetic variables were encoded as discrete numerical indicators prior to model fitting, using simple integer encoding schemes appropriate to the number of genotype categories for each variant.

Model performance was evaluated using a stratified 5-fold cross-validation scheme to reduce the risk of unstable estimates in this relatively small dataset (n = 45). For each neurological manifestation, we built an independent, symptom-specific prediction model. The data were randomly partitioned into five folds of approximately equal size while preserving the class proportions in each fold (StratifiedKFold, n_splits = 5, shuffle = True, random_state = 42). In each fold, the Balanced Random Forest classifier was trained on 80% of the data (four folds) and tested on the remaining 20% (one-fold). For every phenotype, we computed accuracy, precision, recall, F1-score, and the area under the ROC curve (AUC), which is less sensitive to class imbalance; AUC was only calculated for folds in which both classes were present in the test set. We report the mean ± standard deviation of these metrics across the five folds and additionally monitored training accuracy to assess potential overfitting, using cross-validated test metrics to evaluate generalization performance. We repeated this cross-validation analysis several times and obtained very similar performance estimates, indicating stability with respect to the specific train–test partitioning. We did not further increase the number of repetitions because the analysis was conducted separately for eleven different neurological symptoms, and additional repetitions would have resulted in a substantial increase in computation time.

To assess the reliability of the predicted probabilities, we applied post hoc probability calibration using isotonic regression [17] and Platt scaling [18], performed within the same 5-fold cross-validation framework. For each symptom, raw BRF probabilities were first generated on the held-out fold and subsequently calibrated using both methods. Discrimination was quantified using AUC for raw and calibrated probabilities, while calibration accuracy was evaluated using the Brier score [19].

Feature importance for each model was quantified using both cross-validated permutation importance [20] and the mean decrease in Gini impurity [20], yielding ranked estimates of the genetic attributes most strongly associated with each neurological outcome. These analyses were then repeated on the full dataset, and a stability index was computed to assess how consistently each predictor appeared among the top-ranked features across folds and models, allowing us to identify variables reproducibly implicated in neurological vulnerability.

The analytical framework followed Breiman’s Random Forest methodology [20] and the predictive-modeling principles outlined by Kuhn and Johnson [21]. The objectives of this analytical framework were two-fold:

(1) to train predictive models capable of estimating the likelihood of neurological symptoms from detailed genetic descriptors, and,
(2) to identify and rank the genetic predictors most strongly associated with each neurological manifestation. By integrating BRF classification, stability-based feature importance, and calibrated probability estimation, this approach provides a data-driven means of uncovering genotype–phenotype relationships and supports a more mechanistic understanding of neurological vulnerability in Wolfram syndrome.

## 3. Results

### 3.1. Epidemiology

Table 5 shows the demographic profile and genetic confirmation rate of the 45 patients fulfilling the clinical criteria for Wolfram syndrome who were followed longitudinally in Spain between 1998 and 2024. Most patients were of white ethnicity, and genetic confirmation was available in 95.6% of cases. One quarter of patients were born from consanguineous parents, and over one third had affected siblings. The mean age of the surviving cohort in 2024 was 27.5 years, consistent with the natural history reported in previous European cohorts [1,3,22,23].

### 3.2. Cognitive and Neurological Manifestations

Neurological and cognitive manifestations were systematically assessed. Cognitive function was evaluated using the Wechsler Adult Intelligence Scale (WAIS) and, for pediatric participants, the Wechsler Intelligence Scale for Children (WISC). These standardized instruments assess global and domain-specific intelligence (verbal comprehension, working memory, and processing speed). Administration was performed by trained professionals and required approximately 60–90 min. IQ categories were defined as follows:

• Superior intelligence: ≥130.
• High/above average intelligence: 115–129.
• Average intelligence: 85–114.
• Low intelligence (borderline or below average): 70–84
• Intellectual disability: ≤69.

For clinical interpretation, IQ scores were grouped into three bands (high, medium/normal, and low) according to prespecified cut-offs. In the total cohort cognitive assessment was available for 28 patients (62.2%). Among assessed patients, 13 (46.4%) were classified in the high IQ band, 11 (39.3%) in the medium/normal range, and 4 (14.3%) in the low IQ band. At the cohort level, this corresponds to 13/45 (28.9%) high IQ, 11/45 (24.4%) medium/normal IQ, and 4/45 (8.9%) low IQ; cognitive data were unavailable for 17/45 patients (37.8%), and no imputation was performed. The low-IQ band comprised low and borderline scores and included one individual meeting criteria for intellectual disability attributable to congenital cytomegalovirus infection rather than Wolfram syndrome. In a sensitivity analysis excluding this case, the proportion in the low IQ band was 3/27 (11.1%).

Neurological examination systematically covered brainstem, cerebellar, cortical, and peripheral functions, including gait instability, dysmetria, dysphagia, absence of gag reflex, sialorrhea, tremor, and altered deep-tendon reflexes. Each clinical manifestation was encoded as a binary variable (present/absent) for analytical purposes.

**Figure 2:**
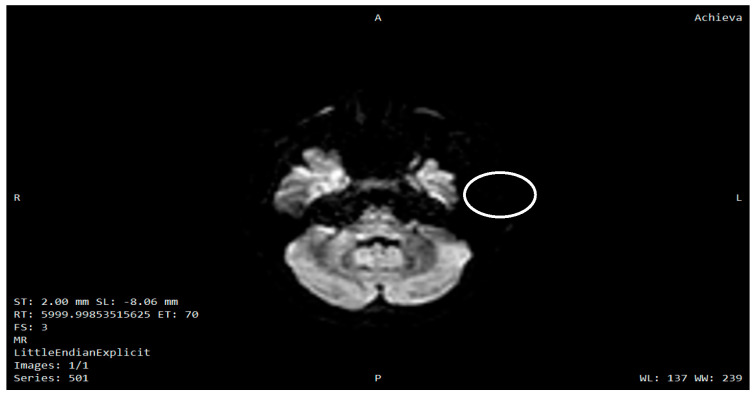
Axial diffusion-weighted MRI showing marked involvement of the pons (circled) in a patient with genetically confirmed Wolfram syndrome. This brainstem lesion pattern is characteristic of the neurodegenerative process described in the disease. A = anterior; P = posterior; R = right; L = left.

**Figure 3:**
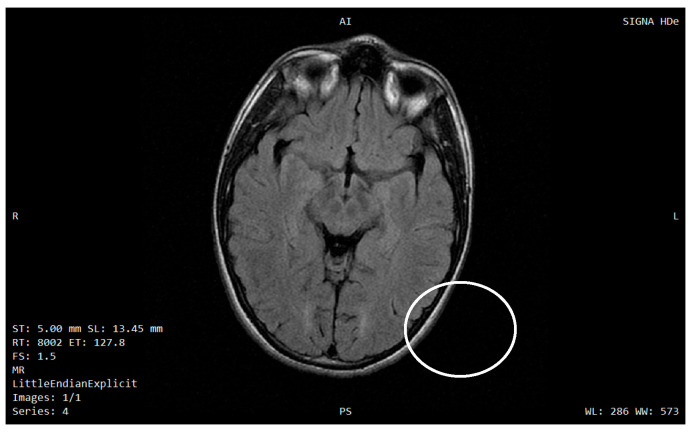
Axial FLAIR MRI showing supratentorial white-matter hyperintensities (circled) in a patient with genetically confirmed Wolfram syndrome, consistent with the neurodegenerative pattern described in this condition. A = anterior; P = posterior; R = right; L = left.

Representative brain MRI images (Figure 2 and Figure 3) were included to illustrate the characteristic neurodegenerative pattern observed in Wolfram syndrome, notably pontine atrophy and supratentorial white-matter abnormalities, which align with the clinical phenotype and previously described neuroanatomical vulnerabilities in WFS1-related disease [7,8,9,10].

**Figure 4:**
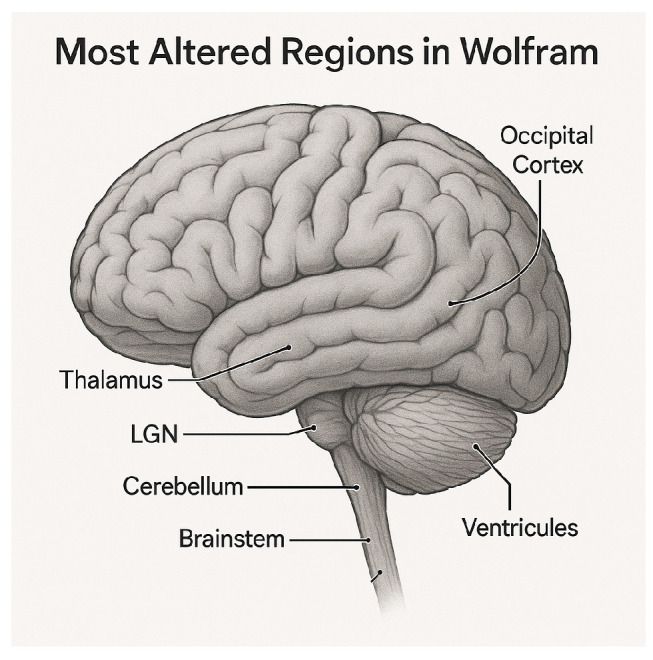
Key neuroanatomical regions most affected in Wolfram syndrome. This illustration highlights the brain structures showing the strongest and most consistent alterations across multimodal MRI metrics, including the thalamus, lateral geniculate nucleus (LGN), brainstem, cerebellum, occipital cortex, and ventricular system. These regions represent the core hubs of neurodegeneration underlying the visual, motor, sensory, and autonomic impairments characteristic of the disease.

Figure 4 shows, according to our analysis, the main areas of the brain that are most impacted in Wolfram syndrome. These include deep sensory and motor control centers (thalamus, LGN), structures important for balance and swallowing (cerebellum and brainstem), the visual cortex, and ventricles. These structures form the core network underlying the multisystem neurological impairment observed in patients. The convergence of abnormalities across multiple systems reflects the exceptional complexity of Wolfram syndrome, a multisystem disorder that affects diverse neural pathways simultaneously.

To avoid confusion, no systematic MRI/DTI study was conducted for this research in this cohort, and the images shown are purely illustrative. A detailed quantitative analysis of MRI/DTI data and their correlation with the different neurological disorders in Wolfram syndrome is beyond the scope of the present work and will be addressed in a dedicated future study.

### 3.3. Correlation Structure Among Neurological Manifestations

**Figure 5:**
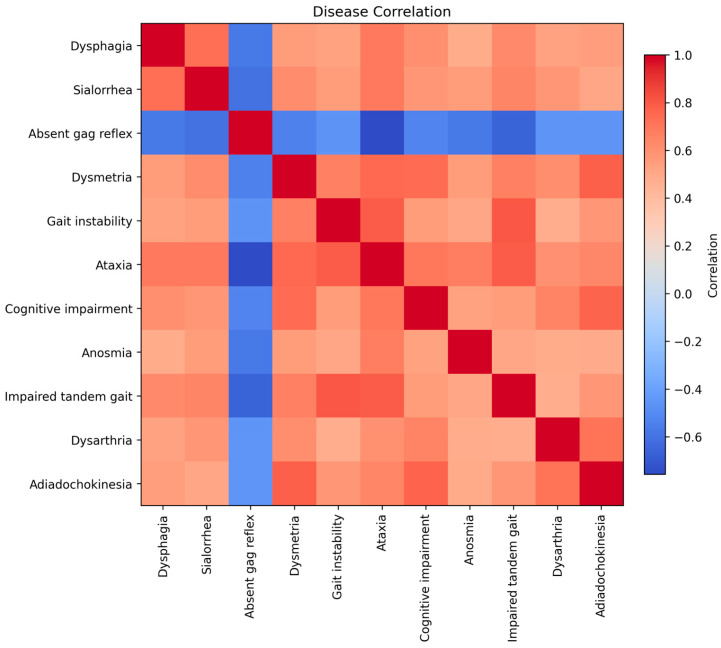
Correlation matrix of neurological manifestations in Wolfram syndrome. Pairwise Pearson correlation coefficients across 11 neurological and cognitive symptoms in the cohort. Most clinical features show moderate-to-strong positive correlations, revealing a cohesive cluster of cerebellar and brainstem dysfunction (including ataxia, gait instability, dysmetria, impaired tandem gait, dysarthria, and adiadochokinesia). Bulbar symptoms such as dysphagia and sialorrhea also correlate closely with these motor findings, reflecting shared pontine and lower brainstem involvement. Cognitive impairment shows parallel associations with the broader neurological deterioration pattern. Anosmia correlates with global disease burden, consistent with diffuse neurodegeneration. In contrast, reflex abnormalities display negative correlations with all other symptoms, suggesting a distinct clinical trajectory or involvement of separate neural pathways. Color intensity reflects the magnitude and direction of each correlation (red = positive; blue = negative).

Figure 5 shows the correlation matrix of neurological manifestations in Wolfram syndrome. The correlation matrix revealed a highly coherent pattern of interdependence among the neurological manifestations of Wolfram syndrome. Most symptoms showed moderate-to-strong positive correlations with one another ( r ≈ 0.47–0.80), indicating a shared underlying neurodegenerative process. A prominent cluster composed of ataxia, gait instability, tandem gait impairment, dysmetria, and adiadochokinesia demonstrated the strongest internal correlations (up to r = 0.81), consistent with a common cerebellar and cerebellar–brainstem origin. Dysphagia and sialorrhea also correlated strongly with these motor features ( r ≈ 0.53–0.73), reflecting convergent involvement of bulbar and pontine motor pathways. Cognitive impairment showed moderate-to-high correlations with both cerebellar and brainstem signs ( r≈ 0.55–0.76), suggesting that supratentorial dysfunction evolves in parallel with infratentorial neurodegeneration rather than as an isolated deficit. Anosmia also correlated with global neurological severity ( r≈ 0.48–0.67), supporting its role as a marker of diffuse neurodegeneration rather than a focal sensory deficit. In contrast, reflex abnormalities exhibited consistently negative correlations with the remaining neurological variables ( r≈ −0.45–0.76). This inverse pattern suggests that reflex impairment may follow a distinct clinical trajectory, potentially reflecting different neural pathways or a biphasic evolution of reflex responses across disease stages. These findings indicate that most neurological manifestations in Wolfram syndrome cluster within a unified brainstem–cerebellar degenerative process that also extends to supratentorial regions, whereas reflex abnormalities may represent an anatomically and clinically distinct dimension of disease involvement.

### 3.4. Genotype–Phenotype Correlation Analysis

A comprehensive correlation analysis was conducted to explore associations between genetic variables and the neurological manifestations observed in the cohort. The strongest correlations identified across all symptom–gene pairs are summarized in Supplementary Table S1. Overall, the results revealed a clear gradient of genotype–phenotype coupling, with cerebellar and gait-related symptoms demonstrating the highest degree of genetic influence.

Ataxia exhibited the strongest associations, with correlation coefficients exceeding 0.54 for multiple genetic predictors, including Wolframin_Class, Mut12_Exon_Class, and interaction terms such as Prod_wm12, Prod_wmg, and Prod_mgm12. These interaction features represent combined effects of wolframin protein dysfunction, mutation location, and zygosity. Their strong correlations with ataxia indicate that cerebellar dysfunction in Wolfram syndrome is closely linked to the severity and structural configuration of WFS1 variants. Similar patterns were observed for tandem gait impairment, gait instability, dysmetria, and adiadochokinesia, all of which displayed correlations between 0.50 and 0.60 with the same set of genetic interactions. These symptoms form a coherent cerebellar–brainstem motor cluster and consistently aligned with genetic predictors reflecting reduced or misfolded wolframin protein. The convergence of findings across multiple motor phenotypes suggests that cerebellar pathways possess the highest genetic penetrance among neurological manifestations in WFS1-related disease.

Cognitive impairment also showed moderate associations ( r ≈ 0.45–0.50) with interaction terms and wolframin functional class, indicating that supratentorial dysfunction progresses partly in parallel with the overall genetic burden. Although not as strongly linked to genotype as cerebellar signs, the magnitude of the correlations supports the notion that cognitive decline in Wolfram syndrome occurs in the context of widespread neurodegeneration influenced by the underlying genetic severity. In contrast, bulbar symptoms such as dysphagia, sialorrhea, and absence of gag reflex demonstrated weaker correlations, generally below 0.35. These manifestations likely reflect a multifactorial process combining genetic vulnerability with downstream neurodegenerative changes in pontine and medullary pathways. Their lower genotype–phenotype coupling is consistent with the machine learning results, in which these symptoms showed only moderate predictability.

The correlation structure indicates that the genetic architecture of WFS1 is most strongly reflected in cerebellar and gait-related motor impairment, while other neurological domains show more heterogeneous relationships. These findings support a model in which the severity and configuration of WFS1 mutations—particularly exon combinations and interaction effects—drive a distinct pattern of cerebellar vulnerability that emerges as the most genetically penetrant neurological phenotype in Wolfram syndrome.

As result of the correlation analysis, Table 6 shows the overall impact of each genetic or molecular attribute across multiple neurological disorders in WS. Across all phenotypic categories, the multimodal prod_ predictors demonstrated the most extensive pattern of associations. Attributes such as prod_wmg, prod_wm12, prod_mgm12, and prod_mgm2 were linked to 6–11 distinct disorders, representing the widest cross-phenotype coverage in the dataset. These variables exhibited high mean absolute correlation coefficients (0.37–0.43) and maximal correlations ranging from 0.52 to 0.57, indicating strong effect sizes. Their recurrent presence across clinically disparate outcomes suggests that the prod_ features capture shared variance underlying global disease burden, likely integrating multiple biological or imaging-derived components that reflect system-level neurodegeneration characteristic of Wolfram syndrome. Genetic classification features, including Mut12_exon_class, wolframin_class, and Genetic_Condition, also showed substantial cross-phenotype involvement, with associations spanning 7–9 disorders and maximum correlation values of 0.51–0.55. These results confirm that genotypic architecture exerts a significant influence on the clinical variability of Wolfram syndrome. However, the breadth and magnitude of these associations were consistently lower than those observed for the multimodal prod_ predictors, suggesting that genetic class alone does not fully account for the multisystem clinical expression of the disorder. In contrast, mutation-specific metrics (e.g., Type_mut2_exon, mut2_protein_class, Type_mut1_exon) exhibited more restricted phenotypic coverage, being associated with only 1–3 disorders and showing lower maximal correlations.

Important to note is that Random Forest feature importance may prioritize genetic variables with low linear correlation because tree-based models capture non-linear interactions and subgroup-defining splits; therefore, importance rankings should not be interpreted as direct measures of association.

### 3.5. Key Clinical-Genetic Findings in Wolfram Syndrome

Neurological manifestations were present in nearly all individuals, with the exception of three younger patients (mean age 14 ± 1.63 years) who showed no detectable neurological involvement at the time of assessment. Cognitive function was largely preserved: 46.6% of patients demonstrated high or superior IQ, 40% showed normal IQ, 11.1% had low IQ, and only one patient (2.2%) met criteria for intellectual disability.

Table 7 summarizes the clinical–genetic characteristics across neurological symptoms. The most prevalent manifestations were absence of gag reflex (67%), gait instability (64%), and dysphagia (60%), reflecting the early and prominent involvement of brainstem and cerebellar motor–autonomic circuits. Additional symptoms such as anosmia (56%), tandem gait impairment (62%), dysarthria (54%), and adiadochokinesia (59%) were also frequent, confirming the multisystem nature of neurological dysfunction in Wolfram syndrome.

Age-of-onset patterns indicate a progressive sequence of neurological involvement. Early manifestations (≈23–24 years)—including dysphagia, sialorrhea, absent gag reflex, and dysmetria—predominantly reflect brainstem and early cerebellar dysfunction. Motor-coordination deficits such as gait instability, ataxia, dysarthria, anosmia, and adiadochokinesia emerged later (≈26–27 years), while cognitive decline occurred last (29.9 ± 12.2 years). This temporal gradient aligns with known neurodegenerative progression in WFS1-related disease.

A moderate sex imbalance was observed across most symptoms, with males representing 60–67% of affected individuals despite accounting for only 55% of the cohort overall. The more balanced distribution observed for the absent gag reflex (53% male) suggests that certain deficits may be less sex-dependent.

Genetic analysis showed that homozygosity for pathogenic WFS1 variants was overrepresented across all symptom categories (≥62%), reaching the highest levels in cognitive decline (81%), anosmia (78%), and adiadochokinesia (82%). The truncating exon 4 c.409_424dup16 (Val142fsX110) variant was the most frequently implicated mutation, appearing across nearly every neurological phenotype, including dysphagia, dysmetria, tandem gait impairment, dysarthria, and adiadochokinesia. Additional variants, such as Trp371X, Val142fs251, and ex8 c.2206G>A, contributed to phenotype-specific presentations, particularly anosmia and dysarthria.

Across all symptoms, the wolframin Type 0 class—corresponding to complete loss of functional protein—was strongly associated with neurological severity, with prevalence ranging from 66% to 83%. Symptoms with the highest Type 0 frequency (ataxia, dysmetria, cognitive decline, anosmia, adiadochokinesia) correspond to circuits known to be highly sensitive to ER-stress dysregulation and calcium imbalance, supporting a unified mechanistic model of selective neuronal vulnerability.

This analysis delineates a clear pattern of genotype-guided neurological progression in Wolfram syndrome:

• Early and highly prevalent motor–autonomic deficits (dysphagia, sialorrhea, absent gag reflex, dysmetria)
• Intermediate cerebellar and motor-coordination involvement (gait instability, ataxia, dysarthria, tandem gait impairment, anosmia)
• Later cognitive decline, associated with high rates of homozygosity and severe protein-loss variants

The predominance of truncating mutations—especially Val142fsX110—and the strong correlation with Type 0 wolframin underscore the central pathogenic role of complete protein loss in driving the severity, distribution, and timing of neurological involvement in Wolfram syndrome. This pattern demonstrates a progressive sequence of neurological involvement, with a slight male predominance, beginning with motor and autonomic manifestations and advancing to sensory and cognitive impairment over time.

### 3.6. Machine Learning Model Performance and Feature Importance

Table 8 summarizes the performance of the genotype-based prediction models across all neurological symptoms using repeated stratified cross-validation (20 repetitions × 5 folds). For each outcome, the table reports prevalence; training accuracy; and the cross-validated mean accuracy, AUC, precision, recall, and F1-score (± SD), together with the aggregated confusion matrix derived from all resampling iterations. These results provide a comprehensive and robust view of the discriminative capacity of the models under repeated data perturbations.

Model performance varied widely across symptoms, showing both areas of moderately reliable predictability and outcomes for which the genetic and interaction features offer limited discriminative power. Among all symptoms, gait instability, ataxia, and absent gag reflex showed the strongest performance, with mean AUC values typically around 0.72–0.84, accuracies around 0.66–0.74, and relatively coherent F1-scores. These symptoms share anatomical and functional substrates involving cerebellar and brainstem pathways—structures known to be affected in Wolfram syndrome—which may partly explain their comparatively stronger genotype–phenotype coupling.

In contrast, symptoms such as dysphagia, anosmia, dysarthria, and tandem gait abnormalities exhibited more modest discrimination, with mean accuracies in the 0.55–0.65 range and lower AUC values (generally 0.60–0.65). These outcomes also showed larger standard deviations across resampling iterations, highlighting their sensitivity to specific train–test partitions in this small and heterogeneous cohort. The lower precision and F1-scores for symptoms like dysarthria further illustrate the challenges posed by class imbalance and limited signal strength in these phenotypes.

These results indicate that while a subset of neurological manifestations—particularly those with clearer structural involvement—show moderate and relatively stable genotype-based predictability, many models exhibit substantial variability and limited robustness. Consequently, these metrics should be interpreted as exploratory rather than definitive indicators of generalization performance, reflecting both the multisystemic complexity of Wolfram syndrome and the constraints imposed by sample size.

Table 9 presents a summary of the calibration and discrimination performance across the neurological symptoms associated with Wolfram syndrome. The results demonstrate distinct patterns of predictability that reflect underlying genotype–phenotype coupling. Overall, raw Random Forest probabilities exhibited limited calibration accuracy, which is expected in rare-disease datasets of small size and heterogeneous clinical expression. However, both isotonic and Platt calibration consistently and substantially improved model performance across all symptoms. Isotonic calibration provided the most reliable probability estimates (lowest Brier scores), whereas Platt scaling often produced marginally higher discrimination (AUC).

This analysis can also be performed using other types of classifiers, such as logistic regression, XGBoost, and others, as well as alternative evaluation metrics like LOOCV. However, the aim of this article is not to design an optimized classifier that addresses the challenges inherent to an ultrarare disease, but rather to demonstrate that the proposed methodology is useful for generating medical hypotheses and improving our understanding of this syndrome.

Nevertheless, as an illustration in the case of dysphagia, we benchmarked two supervised classifiers—Logistic Regression and XGBoost—using accuracy, balanced accuracy, F1-score for the positive class, and ROC-AUC as evaluation criteria, without performing calibration analysis. Logistic Regression yielded the most consistent performance, with LOOCV accuracy of 0.60, balanced accuracy of 0.59, F1-score of 0.65, and AUC of 0.56. XGBoost achieved a comparable F1-score (0.66) but showed markedly lower discrimination, with accuracy of 0.56, balanced accuracy of 0.52, and AUC of 0.50. These findings indicate that dysphagia is a particularly challenging phenotype to model, with weak separability between affected and non-affected individuals and limited benefit from more complex non-linear classifiers.

**Figure 6:**
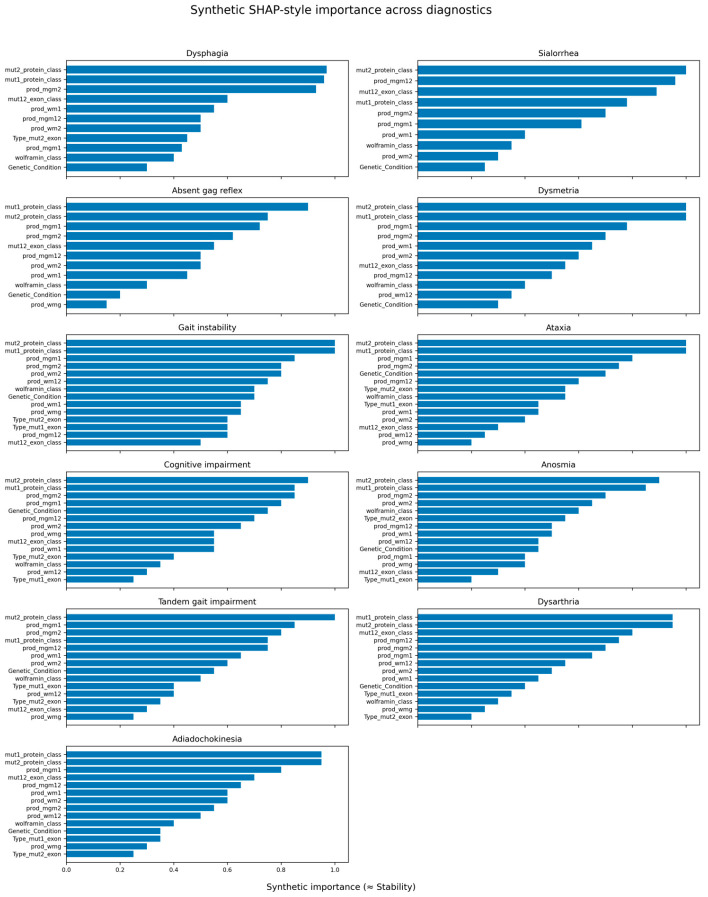
SHAP-based feature-importance patterns across neurological manifestations in Wolfram syndrome indicate a predominantly protein-class–driven architecture. Across almost all phenotypes, Mut1_Protein_Class and Mut2_Protein_Class are the main determinants, with Prod_mgm terms providing secondary refinement and Prod_wm, exon-level (Mut12_Exon_Class), and Genetic_Condition variables contributing only modest additional effects. Dysphagia, dysmetria, ataxia, cognitive impairment, anosmia, dysarthria, adiadochokinesia, and tandem gait impairment all show this consistent dominance of protein-class features, with Prod_mgm interactions capturing phenotype-specific vulnerability (e.g., brainstem in dysphagia, cerebellar timing in dysmetria and ataxia) and only minor modulation by exon-level or broader genetic-context variables.

Table 10 shows a summary of the dominant, secondary and marginal predictors across all clinical phenotypes. A more comprehensive analysis for each neurological condition is provided in the supplementary material (Tables S2–S12). Figure 6 displays SHAP summary diagrams illustrating the stability and relative contribution of genetic predictors across the full spectrum of neurological symptoms in Wolfram syndrome.

The stability index in this analysis was defined as the agreement between cross-validated and full-model feature rankings, calculated as the normalized inverse distance between the two ranks:

$$St=1-\frac{\left|r_{CV}-r_{FULL}\right|}{r_{max}-1}$$

where $r_{CV}$ is the feature’s rank in cross-validated importance, $r_{FULL}$, the feature’s rank in the full-model importance and $r_{max}$, the maximum possible rank (i.e., number of features). Values of St closer to 1 indicate highly concordant rankings and therefore more robust, reproducible predictors.

Based on the Stability index, we have defined the following categories for the features:

• Dominant: ST≥0.85.
• Consistent Secondary: ST∈[0.60–0.85].
• Weak: ST<0.6.

Across all phenotypes studied, Mut1_Protein_Class and Mut2_Protein_Class consistently emerge as the strongest predictors. This pattern indicates that the protein-level consequences of each mutation—loss of wolframin, reduced production, or misfolding—are the primary determinants of neurological expression in Wolfram syndrome. Whether individuals present with dysphagia, dysmetria, ataxia, anosmia, dysarthria, tandem gait impairment, adiadochokinesia, or cognitive involvement, the decisive factor is the functional state of wolframin produced by each allele.

Phenotypes that rely heavily on precise motor coordination (dysmetria, ataxia, adiadochokinesia, tandem gait impairment) show a particularly marked dependence on Mut1_Protein_Class and Mut2_Protein_Class. This makes sense biologically: cerebellar and motor brainstem circuits are highly sensitive to disruptions in cellular stress management and calcium homeostasis—key processes in which wolframin plays an essential role. Symptoms like dysphagia and absent gag reflex, which reflect brainstem reflex integration, also display a strong protein-driven signature, reinforcing the idea that wolframin insufficiency directly compromises the survival or function of neurons in these pathways.

Clinically, this suggests that certain mutations exert their influence not through direct linear effects on individual symptoms, but by defining patient subgroups with distinct clinical trajectories and symptom clusters, which Random Forest models capture as high-importance features.

A consistent secondary layer of predictors is formed by the Prod_mgm interactions (Prod_mgm1, Prod_mgm2, Prod_mgm12). By combining Genetic_Condition with exon-level descriptors (Type_mut1_exon, Type_mut2_exon, Mut12_Exon_Class), these interaction terms capture how zygosity modifies the impact of specific exon mutations. Their presence across nearly every phenotype suggests that the way mutations combine across alleles—and the exons they affect—modulates symptom severity, especially when both alleles contribute functionally distinct defects. This helps explain why some compound-heterozygous configurations exhibit more severe or more widespread neurological symptoms than others with similar protein-class outcomes.

The prod_wm interaction terms (Prod_wm1, Prod_wm2, Prod_wm12) introduce small but reproducible contributions. These variables represent interactions between Wolframin_Class (Type 0–3) and exon-based mutation classes. Their consistent but modest influence suggests that the pattern of wolframin production interacts subtly with the structural location of mutations, forming a refinement layer that might shape how early or how severely certain circuits are affected. This is particularly evident in phenotypes involving fine motor control and timing, where subtle shifts in functional reserve may be clinically noticeable.

Exon-derived features themselves (especially Mut12_Exon_Class) show phenotype-specific influence—moderate in some (e.g., Dysmetria, Dysarthria) and marginal in others. This suggests that whether both mutations affect the same exon or different exons can alter the functional combined effect of the two alleles, but usually only after the major protein-class consequences are accounted for. Meanwhile, Genetic_Condition and Wolframin_Class on their own display very limited predictive value, showing that broad categories add little once detailed mutation classes and interactions are included.

## 4. Discussion

This study provides new insights into the genotype–phenotype relationships in Wolfram syndrome (WS) by integrating comprehensive genetic characterization with machine learning-based predictive modeling. Our findings demonstrate that functional impairment of wolframin, rather than mutation type alone, is the strongest determinant of neurological severity and disease progression.

### 4.1. Genetic Mechanisms and Phenotypic Expression of Neurological Disorders

Patients carrying biallelic truncating variants—particularly the ex4 c.409_424dup16 (Val142fsX110) mutation—showed the most severe and early-onset neurological manifestations, including dysphagia, dysmetria, and gait instability [6,24,25]. These results align with previous studies reporting progressive brainstem and cerebellar atrophy as major hallmarks of WS [7,8,9,10]. The predominance of Class 0 wolframin, defined by complete absence of protein production, supports a mechanistic model in which ER stress and calcium dysregulation trigger selective neuronal vulnerability. The temporal pattern observed—motor and autonomic dysfunction in the early 20s, followed by sensory and cognitive decline—suggests a sequential neurodegenerative cascade consistent with the progressive nature of the syndrome.

Interpretation of cognitive findings in Wolfram syndrome requires particular caution, as several multisystemic features may influence test performance independently of primary cortical dysfunction. Progressive visual loss and sensorineural hearing impairment can affect tasks requiring sustained attention, processing speed, or working memory. Visual impairment may bias visually mediated cognitive tests, and the use of vision-independent measures has been proposed to reduce this effect in visually impaired populations [26]. Likewise, hearing loss has been associated with poorer performance in memory and executive-function domains, potentially confounding cognitive assessment in multisensory disorders [27]. Additional factors—including fatigue, sleep–wake disruption, and psychiatric comorbidities such as anxiety, depression, or OCD-spectrum symptoms—may further impact cognitive performance [28,29]. Metabolic instability, particularly episodes of acute hypoglycaemia and glycaemic variability, can also introduce short-term fluctuations in cognition; hypoglycaemia can transiently impair multiple domains, and glycaemic variability has been linked to cognitive variability in diabetes cohorts [30,31]. Collectively, these features may partially mask or mimic early cognitive decline. Therefore, the IQ distribution described here should be interpreted within the broader multisystemic context characteristic of WFS1-related disease [26,27,28,29,30,31].

In our cohort, cognitive impairment was generally recognized later than motor and autonomic symptoms, consistent with previous reports describing relatively preserved global cognition in many children and adolescents with Wolfram syndrome despite early neurological and structural abnormalities [10,28,32,33]. Several mechanisms may contribute to this pattern. First, cognitive reserve—supported by premorbid abilities and educational attainment—may help maintain functional performance despite accumulating neuropathology, delaying the clinical expression of cognitive deficits [34,35,36]. Second, supportive family, school, and healthcare environments, together with compensatory strategies, may mitigate the functional impact of early cognitive changes and postpone formal recognition [37,38]. Given the sample size and design of the present study, we cannot disentangle the relative contribution of these factors. Longitudinal studies with standardized cognitive and functional assessments will be important to address these questions more directly.

### 4.2. Predictive Modeling and Variable Importance

With respect to model performance, neurological manifestations with clearer cerebellar or brainstem substrates showed the strongest genotype-based predictability. Across the repeated cross-validation results summarized in Table 8, ataxia and gait instability yielded the highest discrimination (mean AUC ≈ 0.75–0.84) and comparatively stronger accuracy and F1-scores, followed by the absence of gag reflex. These manifestations map onto neuroanatomical systems—particularly cerebellar and brainstem pathways—that are known to be preferentially affected in Wolfram syndrome, suggesting tighter and more consistent genotype–phenotype coupling.

In contrast, outcomes such as dysphagia, anosmia, dysarthria, and impaired tandem gait demonstrated only modest discrimination (AUC ≈ 0.60–0.65) and lower accuracy, together with larger standard deviations across the repeated folds. These symptoms are clinically heterogeneous and arise from multisystem mechanisms extending beyond focal brainstem–cerebellar pathways, which likely contributes to the weaker, more unstable predictive performance observed in this small cohort.

Given that Table 8 consolidates outcomes from multiple repetitions of stratified 5-fold cross-validation, the mean ± SD estimates provide a direct measure of model stability. The substantial variance observed for dysarthria, anosmia, and adiadochokinesia reflects the strong influence of fold composition on performance metrics, emphasizing the methodological challenges of modeling multisystem phenotypes in a 45-patient cohort. These results show that while certain neurological manifestations exhibit moderately stable genotype-based predictability, many models remain highly sensitive to data partitioning and should be interpreted as exploratory. Larger, harmonized multicenter datasets will be essential to validate these genotype–phenotype relationships and improve predictive robustness.

Three predictability classes emerged from this analysis:

1. High Predictability Class: Ataxia, Gait Instability and Absent Gag Reflex. These symptoms showed the highest raw AUC values and exceptional calibrated performance (AUC > 0.95). Their strong predictability aligns with well-established cerebellar and brainstem involvement in Wolfram syndrome, suggesting direct and consistent genetic influence.
2. Intermediate Predictability Class: Dysmetria, Cognitive Impairment, Anosmia, Adiadochokinesia and Impaired Tandem Gait. These symptoms demonstrated moderate raw discrimination but substantial calibration gains. Their multifactorial nature and involvement of broader neural systems likely dilute genotype–phenotype coupling, yet calibration reveals meaningful underlying structure.
3. Low Predictability Class: Dysphagia, Sialorrhea, Dysarthria. These symptoms exhibited the weakest raw predictive performance, with calibration improving results but not to the level of more genetically constrained manifestations. Their heterogeneity and dependence on multiple physiological pathways limit purely genetic predictability.

These results indicate that while raw machine learning predictions are insufficient for clinical use, post hoc calibration uncovers robust and biologically meaningful patterns. These results support the exploratory use of machine learning in Wolfram syndrome and motivate future multimodal approaches integrating genetics, MRI, and longitudinal follow-up to enhance interpretability and predictive performance.

With respect to model importance and the generation of medical hypotheses, the consistency with which protein-level mutation classes emerged as the top predictors across all analyses reinforces their central role in driving neurological vulnerability and provides a robust, biologically grounded basis for interpreting genotype–phenotype relationships in Wolfram syndrome. Across all phenotypes studied, Mut1_Protein_Class and Mut2_Protein_Class uniformly dominated feature importance, indicating that the functional consequences of each allele—loss of wolframin, reduced production, or misfolding—are the principal determinants of neurological expression, regardless of the specific neural circuit involved. This unifying pattern supports the view that wolframin deficiency constitutes a shared upstream mechanism underlying dysphagia, dysmetria, ataxia, anosmia, dysarthria, tandem gait impairment, adiadochokinesia, and cognitive involvement. Motor coordination phenotypes (dysmetria, ataxia, adiadochokinesia, and tandem gait impairment) showed particularly strong dependence on protein-class variables, which aligns with the high vulnerability of cerebellar and cerebello-thalamo-cortical systems to disturbances in ER stress regulation and calcium homeostasis—core functions of wolframin. Similarly, brainstem reflex symptoms (dysphagia, absent gag reflex) displayed a clear protein-driven signature, consistent with selective susceptibility of motor nuclei and sensorimotor integration pathways.

A consistent secondary layer of predictors was formed by the Prod_mgm interactions (Prod_mgm1, Prod_mgm2, Prod_mgm12), which combine Genetic_Condition with exon-level descriptors (Type_mut1_exon, Type_mut2_exon, Mut12_Exon_Class). These interactions capture how zygosity and exon location shape the phenotypic impact of each mutation. Their recurrence across symptoms suggests that the configuration of mutations across alleles—particularly when involving different exons—modulates the severity and extent of neurological deficits, helping explain why compound-heterozygous genotypes with similar protein-level consequences may nonetheless show different clinical profiles.

The Prod_wm interaction terms (Prod_wm1, Prod_wm2, Prod_wm12), although modest in magnitude, were reproducible across models. These features represent interactions between Wolframin_Class (Type 0–3) and exon-level mutation classes, implying that subtle differences in wolframin production (e.g., partial vs. misfolded protein) interact with mutation location to fine-tune phenotype expression—particularly in motor systems where temporal precision is critical.

Exon-derived predictors, especially Mut12_Exon_Class, showed phenotype-specific contributions, being moderately informative for coordination-related symptoms but less relevant for others. This pattern indicates that whether both mutations fall in the same exon or in different exons alters the combined functional effects of the alleles, but typically only after accounting for the dominant protein-level consequences. In contrast, broad variables such as Genetic_Condition and Wolframin_Class alone had limited predictive value, demonstrating that coarse genotype categories add little once detailed mutation classes and their interactions are included.

These patterns allow for a symptom-specific interpretation of how different genetic mechanisms shape the neurological presentation of Wolfram syndrome, as summarized below for each phenotype category:

• Motor coordination phenotypes (Dysmetria, Ataxia, Tandem Gait Impairment, Adiadochokinesia): Strong protein dependence suggests that cerebellar and cerebello-thalamo-cortical loops are highly vulnerable to wolframin deficits. Prod_mgm signals imply that mutation configuration across alleles affects the degree of dysfunction.
• Brainstem reflex phenotypes (Dysphagia, Absent Gag Reflex). Protein-level effects dominate, consistent with selective vulnerability of motor nuclei and sensory integration centers. Prod_mgm terms suggest that some exon combinations might exacerbate these deficits.
• Sensory phenotypes (Anosmia). Strong protein effect, but overall lighter interaction structure, consistent with olfactory circuits being affected mainly by global wolframin insufficiency.
• Speech-related phenotypes (Dysarthria, Sialorrhea). Protein-driven with coherent Prod_mgm contributions, likely reflecting combined cerebellar, corticobulbar, and brainstem involvement.
• Cognitive Impairment. Still strongly protein-class driven, with modest contributions from Genetic_Condition and Prod_mgm terms, reflecting more distributed vulnerability across neural systems.

**Figure 7:**
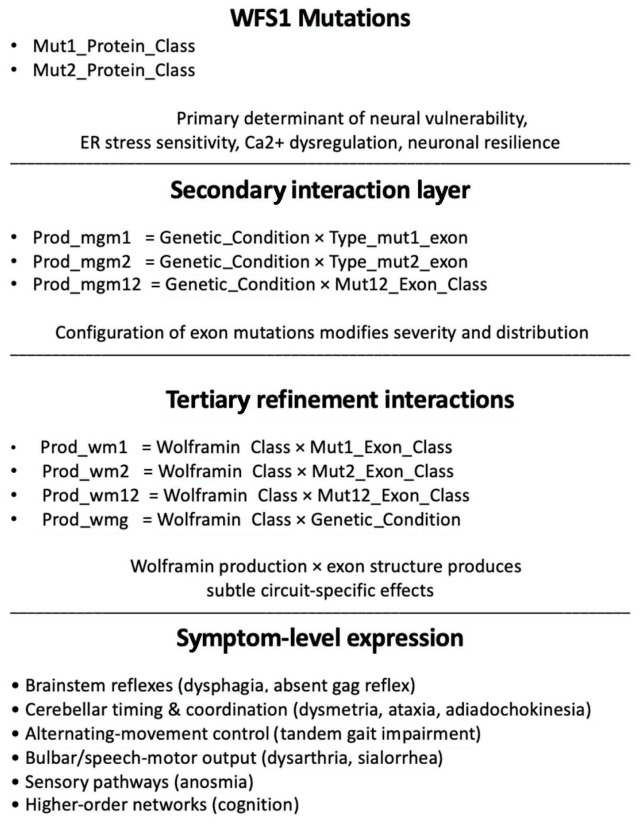
Hierarchical structure of genetic determinants shaping neurological expression in Wolfram syndrome. Across all models, Mut1_Protein_Class and Mut2_Protein_Class dominate feature importance, indicating that wolframin deficiency—through protein loss, reduction, or misfolding—is the main driver of neurological involvement. Prod_mgm interaction terms (Prod_mgm1, Prod_mgm2, Prod_mgm12), which combine zygosity with exon-level structure, form a consistent secondary layer that modulates symptom severity. Smaller but reproducible Prod_wm effects (Prod_wm1, Prod_wm2, Prod_wm12, Prod_wmg) provide finer refinement, capturing subtle interactions between wolframin production type and exon location, and together these layers account for both shared patterns and inter-individual variability in WFS1-related neurological manifestations.

Figure 7 shows the suggested hierarchical structure of the genetic determinants shaping neurological expression in Wolfram syndrome. Mechanistically, the dominant influence of Mut1_Protein_Class and Mut2_Protein_Class suggests a common mechanism: neurological symptoms in Wolfram syndrome arise primarily from the functional loss or misfolding of wolframin, irrespective of the specific neural circuit affected. The consistent presence of Prod_mgm interactions indicates that the combination of mutations across alleles (zygosity × exon structure) modulates severity and contributes to patient-to-patient variability. The smaller but stable Prod_wm contributions show that differences in the type of wolframin produced subtly interact with mutation location to refine symptom expression, especially in cerebellar and motor phenotypes. The neurological variability across Wolfram syndrome is principally determined by protein-level mutation effects, with zygosity–exon interactions providing a modulatory layer and wolframin-production interactions contributing a fine-grained tuning effect.

It should also be emphasized that *mut1* and *mut2* do not represent biologically distinct alleles, as their ordering in the dataset is arbitrary; consequently, differences in feature importance between *mut1_protein_class* and *mut2_protein_class* should not be interpreted as allele-specific dominance, and interpretation should instead focus on the overall functional classification of wolframin and the combined biallelic mutation pattern.

In this context, we use “prediction” in a statistical sense: the models identify genetic variables that are associated with, and can predict the probability of, each neurological phenotype, but they do not imply deterministic or strictly causal genotype–phenotype relationships. These associations should therefore be viewed as predictors of risk and as hypothesis-generating findings that require confirmation in independent cohorts.

### 4.3. Clinical and Translational Implications

The predictive modeling results provide several clinically meaningful insights into the neurological progression of Wolfram syndrome. In terms of model performance, the strongest genotype–phenotype coupling was observed for manifestations with clearer cerebellar or brainstem substrates. Ataxia and gait instability showed the highest discriminative ability (mean AUC ≈ 0.75–0.84) together with comparatively higher accuracy and F1-scores, while absence of gag reflex also demonstrated moderate discrimination. These findings suggest that deficits involving cerebellar–brainstem coordination are among the most tightly linked to the underlying genetic architecture of WFS1-related neurodegeneration and may represent earlier or more genetically driven components of the neurological phenotype.

Symptoms reflecting cerebellar–brainstem integration, such as dysmetria and adiadochokinesia, also exhibited moderate predictive performance (AUC ≈ 0.70–0.72), consistent with the vulnerability of these pathways to wolframin deficiency. In contrast, more heterogeneous or multisystem manifestations, including dysphagia, dysarthria, anosmia, and impaired tandem gait, displayed only modest discrimination (AUC ≈ 0.58–0.65) and greater fold-to-fold variability. These patterns reflect the complex and distributed mechanisms contributing to these symptoms, which likely dilute the signal that can be inferred from genotype alone in a cohort of this size.

From a clinical perspective, these findings highlight the potential value of integrating genotype-based risk prediction into routine care. Beyond motor and brainstem manifestations, circadian rhythm disturbances and psychiatric features represent an additional dimension of disease burden in WS and may require specific chronobiological and psychiatric assessment [39]. Patients carrying genotypes strongly associated with high-risk symptom clusters (e.g., reduced or misfolded protein classes, deleterious zygosity × exon combinations) may benefit from intensified surveillance. Early identification of individuals at increased risk for dysphagia, silent aspiration, or airway compromise—conditions closely linked to morbidity and mortality in Wolfram syndrome—could guide anticipatory interventions such as modified feeding protocols, respiratory monitoring, or early ENT assessment. The model also supports prognostic counseling and helps clinicians prioritize referrals to multidisciplinary care, particularly for patients at risk of rapid neurological deterioration.

From a translational perspective, the study demonstrates that machine learning-derived feature importance profiles provide a robust and reproducible framework for evaluating the pathogenic potential of newly identified WFS1 variants. The consistent dominance of Mut1_Protein_Class and Mut2_Protein_Class underscores that protein-level consequences—loss of function, misfolding, or incomplete production—are central to neurological vulnerability. The stable contribution of interaction terms (Prod_mgm and Prod_wm) further suggests that specific allele combinations modulate symptom severity, supporting the incorporation of epistatic effects into future variant-interpretation guidelines.

Finally, the modeling framework offers a quantitative basis for patient stratification in interventional or neuroprotective trials. By identifying individuals whose genetic profiles predict early or rapidly progressive neurological involvement, trials can incorporate risk-enrichment strategies that increase statistical power and improve the detection of therapeutic effects. More broadly, the model provides clinicians with actionable information that can refine risk stratification, support prognosis, and inform the design of future disease-modifying interventions in Wolfram syndrome.

### 4.4. Current Therapeutic Strategies and Clinical Trials in Wolfram Syndrome

Although no disease-modifying treatment is currently approved, several therapeutic approaches are under investigation. Clinical trials have explored the use of dantrolene (an endoplasmic-reticulum stress modulator), sodium valproate, GLP-1 receptor agonists, and AMX0035 as potential neuroprotective agents.

Symptomatic management—including glycemic control, audiological rehabilitation, ophthalmological monitoring, and treatment of dysphagia—remains essential in current clinical practice. Emerging preclinical therapies targeting ER stress, calcium dysregulation, and mitochondrial dysfunction, as well as gene-based and regenerative strategies, represent promising avenues for future intervention. Incorporating this information provides clinicians with an updated overview of the evolving therapeutic landscape in Wolfram syndrome [3,25].

### 4.5. Limitations and Future Perspectives

This study has several limitations. The most important is the relatively small cohort size ( n=45) and sex imbalance, both inherent to the extreme rarity of Wolfram syndrome. In addition, the cross-sectional design restricts causal inference and does not allow assessment of trajectories or rates of neurological decline. Although the feature-engineering strategy was comprehensive, genotype–phenotype patterns observed in a single national cohort warrant validation in larger and more diverse populations.

Future research should prioritize multicenter, longitudinal studies to confirm the stability and generalizability of these models [6,24]. Integrating multimodal biomarkers such as EEG, quantitative MRI (volumetrics and diffusion imaging), and molecular indicators of ER stress may further clarify how cellular dysfunction driven by WFS1 loss translates into clinical progression. Such multimodal integration is likely to enhance model performance and support the development of clinically deployable AI-based prediction tools. In this article, we provide preliminary evidence by examining the discriminatory power of several genetic attributes across the main neurological disorders. In future work, we will extend this framework to systematically compare the contribution of genetic and MRI-derived features across the full spectrum of Wolfram-related phenotypes.

In this study, each patient’s WFS1 mutations were systematically categorized according to exon location and predicted functional impact. Exon-based variables (mut1_exon_class, mut2_exon_class, and mut12_exon_class) captured whether mutations occurred in exon 4, exon 8, or across both. Protein-level variables (mut1_protein_class and mut2_protein_class) described the functional consequences of each allele, while Genetic_Condition reflected zygosity (homozygous, compound heterozygous, or triple heterozygous). Wolframin production was stratified into four functional classes, ranging from complete loss (class 0) to partial or misfolded production (classes 1 and 2) and autosomal-dominant patterns with preserved protein output (class 3). These structured genetic descriptors made it possible to model allele-interaction effects with precision and to explore epistatic patterns relevant to disease severity.

In interpreting these findings, it is important to acknowledge the risk of overfitting when applying machine learning methods to a small clinical cohort. With few patients and many predictors, the models may capture idiosyncrasies of this dataset rather than stable, generalizable patterns. To limit this risk, we used stratified k-fold cross-validation, kept each subject exclusively in either training or test sets within a fold, and constrained model complexity by limiting tree depth and the minimum number of samples per node. Performance metrics were averaged across folds, and, for each symptom, the stratified 5-fold procedure was repeated 20 times to empirically assess stability. The repeated analysis showed that mean performance changed little, but standard deviations often increased—especially for low-prevalence symptoms—indicating that a single 5-fold split underestimates uncertainty and that several models are sensitive to the specific train–test partitions in this small cohort.

A further limitation is that a fully exhaustive analysis of fold-wise confusion matrices and feature-importance profiles for every predictor and every neurological outcome would require larger, better-balanced cohorts to provide stable and interpretable estimates. We therefore focus on global model performance and on the most robust and biologically plausible patterns of feature relevance, rather than on fine-grained differences between disorders. Even with these precautions, some optimistic bias cannot be excluded, and the models should be regarded as exploratory and hypothesis-generating. External validation in larger, independent Wolfram syndrome cohorts will be essential to confirm the robustness and clinical utility of the reported associations and feature-importance patterns.

This work demonstrates that functional classification of wolframin and allele-interaction modeling can predict several neurological outcomes in Wolfram syndrome, despite the constraints of a rare-disease cohort. By linking molecular dysfunction to clinical expression through machine learning methods, this study provides a foundation for precision-medicine strategies in WS and highlights the promise of computational tools for stratifying risk, guiding surveillance, and informing future interventional trials.

## 5. Conclusions

In this study, we integrated detailed genetic characterization with machine learning models to clarify genotype–phenotype relationships in Wolfram syndrome. Our results show that functional impairment of wolframin is the strongest determinant of neurological severity, with protein-class variables (Mut1_Protein_Class and Mut2_Protein_Class) consistently emerging as the dominant predictors across all symptoms. These mutations do not act via straightforward linear effects on single symptoms but instead define subgroups of patients with different clinical courses and symptom patterns, which are captured by Random Forest as highly important features.

Neurological manifestations with well-defined cerebellar or brainstem substrates—particularly ataxia, gait instability, and absence of gag reflex—showed the highest discriminative performance (calibrated AUC up to 0.97), indicating tight genotype–phenotype coupling.

Interaction effects based on zygosity and exon location provided additional explanatory value, helping account for inter-individual variability within this rare disorder. Clinically, these findings support the use of genotype-based risk stratification to guide surveillance for high-risk complications such as dysphagia and airway compromise and offer a quantitative framework for interpreting newly identified WFS1 variants.

Despite the limitations inherent to a small, cross-sectional cohort, this work demonstrates that combining functional genetic classification with machine learning methods can meaningfully predict neurological outcomes in Wolfram syndrome and provides a foundation for future precision-medicine tools and risk-enriched neuroprotective trial design.

## Figures and Tables

**Table 1:** Genetic status of Spanish patients with Wolfram syndrome. This table summarizes the distribution of WFS1 genotypes in the cohort. Patients were classified as homozygotes, compound heterozygotes, or triple heterozygotes (carriers of two variants on one allele and a third variant on the homologous chromosome). Overall, 48.9% of individuals were heterozygous carriers (compound or triple heterozygotes), reflecting the high allelic diversity characteristic of WFS1-related disorders in this population.

Variable	Categories	Frequency in Cohort
WFS1 genotype	1 = WFS1 homozygote 2 = WFS1 compound heterozygote 3 = WFS1 triple heterozygote (one allele with two mutations + the homologous allele with one mutation)	48.9% heterozygotes

**Table 2:** WFS1 variants identified at the cDNA level. This table lists all nucleotide-level WFS1 variants detected in the cohort, reported separately for each allele. Variants follow HGVS cDNA nomenclature and include missense, nonsense, frameshift, in-frame indels, and splice-related changes across exons 4 and 8 of the WFS1 gene. “Mutation 1” and “Mutation 2” correspond to the two inherited alleles for each individual, and “None” indicates that no coding variant was detected in that allele.

Allele	Variants Identified (c.DNA)
Mutation 1 (Allele 1)	ex4 c.409_424dup16; ex4 c.472 G>A; ex4c.489_424dup; ex4 c.506delA; ex8c.1060_1062delTTC; ex8 c.1096 C>T; ex8 c.1113 G>A; ex8 c.1124 G>A; ex8 c.1230_1233delCTCT; ex8 c.1289 C>A; ex8 c.1558 C>T; ex8 c.1582 T>G; ex8 c.2020 G>A; ex8 c.2051 C>T; ex8 c.2118 C>A; ex8 c.2206 G>A; ex8 c.2209 G>A; ex8 c.2564 C>G; ex8 c.873 C>A; ex8 c.963_966del4; ex8 c.977 C>T
Mutation 2 (Allele 2)	ex4 c.409_424dup16; ex8 c.1113 G>A; ex8 c.1230_1233del; ex8 c.1230_1233delCTCT; ex8 c.1329 C>G; ex8 c.1340 T>C; ex8 c.1456_1457insT; ex8 c.1462_1463ins12; ex8 c.1511 C>T; ex8 c.1525_1538del15; ex8 c.1558 C>T; ex8 c.1582 T>G; ex8 c.1612 T>C; ex8 c.2020 G>A; ex8 c.2118 C>A; ex8 c.2206 G>A; ex8 c.2206 G>C; ex8 c.2209 G>A; ex8 c.2257 G>T; ex8 c.854 G>T; ex8 c.873 C>A; ex8 c.873C>A; ex8 c.963_966del; ex8 c1463_1474

**Table 3:** WFS1 mutations identified at the protein level. This table lists all WFS1 protein alterations detected in the cohort, reported separately for each allele. Variants include missense, nonsense, frameshift, splice-related changes, and in-frame deletions affecting different regions of the wolframin protein. Allele 1 and Allele 2 correspond to the two independent WFS1 alleles carried by each individual, and “None” indicates absence of a detectable coding variant in that allele.

Allele	Variants Identified (WFS1 Protein)
Mutation 1 (Allele 1)	None; Ala326Val; Ala684Val; Arg375His; Gln366X; Gln520X; Glu158Lys; Glu169GlyfsX2118; Glu674Arg; Glu737Lys; Gly736Arg; Gly736Ser; His322fsX; Phe354del; Phe354fs*; Phe854del; Phedel; Ser430X; Ser855fs*; Trp371X; Tyr706X; TyrX*; Val142Glyfs*118; Val142Glyfs*X; Val142fs*; Val142fs251*; Val142fsX; Val142fsX110; Val412Serfs*29; Val412SerfsX29; Val412Serfs*29; Y528D
Mutation 2 (Allele 2)	None; Arg285Leu; Gln486Leufs*57X; Gln520X; Glu674Arg; Glu737Lys; Glu753X; Gly736Arg; Gly736Ser; His322Thrfs*; Leu447Pro; Phe538Leu; Pro504Leu; Ser443Arg; Trp371X; Tyr706X; Val142fs*; Val142fs110; Val142fs251*; Val142fsX110; Val412Serfs*29; Val412SerfsX; Val491ProinsLeuIleThrVal; Val509_Tyr513del5; Valfs*; Y528D

X denotes a stop codon. In frameshift variants (fs), the asterisks are part of the HGVS protein-variant nomenclature (e.g., fs*29 indicates a frameshift with a premature stop codon 29 amino acids downstream).

**Table 4:** Genetic and interaction variables used in the machine learning model. This table lists all genetic and interaction features included in the predictive models, together with a brief description of their biological or functional relevance. Variable names correspond exactly to those used in the analyses and throughout the manuscript.

Variable	Definition/Description
Mut1_Protein_Class	Protein effect of mutation on allele 1.
Mut2_Protein_Class	Protein effect of mutation on allele 2.
Mut12_Exon_Class	Classification based on whether both mutations are located in the same exon (exon 4, exon 8) or in different exons.
Type_mut1_exon	Type of mutation affecting allele 1 and its corresponding exon.
Type _mut2_exon	Type of mutation affecting allele 2 and its corresponding exon.
Genetic_Condition	Categorical variable indicating zygosity (homozygous, compound heterozygous, triple heterozygous).
Wolframin_Class	Classification of wolframin protein production: Type 0: No protein (premature stop codon).Type 1: ~50% protein, likely non-functional (misfolding).Type 2: Misfolded protein from both alleles. Type 3: Autosomal dominant case, ~50% normal protein production.
Prod_wm1	Interaction term: Wolframin class × Mut1_Exon_Class.
Prod_wm2	Interaction term: Wolframin class × Mut2_Exon_Class.
Prod_wm12	Interaction term: Wolframin class × Mut12_Exon_Class.
Prod_wmg	Interaction term: Wolframin class × Genetic_Condition.
Prod_mgm1	Interaction term: Genetic_Condition × Mut1_Exon_Class.
Prod_mgm2	Interaction term: Genetic_Condition × Mut2_Exon_Class.
Prod_mgm12	Interaction term: Genetic_Condition × Mut12_Exon_Class.

**Table 5:** Demographic and Genetic Characteristics of the Spanish Wolfram syndrome cohort (1998–2024).

Characteristic	*n* (%)/Mean ± SD
Total patients	45
Sex	Male:25 (55.5%) Female: 20 (44.5%)
Genetic confirmation available	43 (95.6%)
Parental consanguinity	12 (26.7%)
Siblings within cohort	17 (37.8%)
Ethnicity	White:39 (86%) Romani: 4 Arab origin: 2
Vital status (2024)	Survivors: 35 Deceased: 10
Age (survivors)	27.5 ± 11.1 years

**Table 6:** Attributes with the strongest global impact across neurological disorders. This table lists the genetic and interaction variables that demonstrated the highest overall influence across the symptom-specific machine learning models. For each attribute, the number of disorders in which it appeared among the top contributors is indicated, along with its mean absolute correlation and maximum absolute correlation across all outcomes. Interaction terms involving wolframin class, mutation exon classification, and genetic condition consistently showed the strongest associations, highlighting the relevance of combined genetic effects in shaping the phenotypic spectrum of WFS1-related disease.

Attribute	No. of Disorders	mean_abs_corr	max_abs_corr
Prod_wmg	7	0.41	0.57
Prod_wm12	8	0.37	0.56
Prod_mgm12	10	0.43	0.55
Mut12_exon_class	9	0.4	0.55
Wolframin_Class	7	0.38	0.54
Prod_mgm2	11	0.42	0.54
Prod_wm1	6	0.38	0.52
Prod_wm2	6	0.38	0.52
Genetic_Condition	9	0.42	0.51
Prod_mgm1	7	0.41	0.47
Type _mut2_exon	3	0.39	0.42
Mut2_protein_class	3	0.35	0.38
Type _mut1_exon	1	0.35	0.35

**Table 7:** Key Clinical-Genetic Findings in Wolfram Syndrome. This table summarizes the main clinical and genetic characteristics associated with each neurological symptom in the cohort. For every outcome, the mean age of onset, proportion of affected males, and percentage of homozygous WFS1 mutations are reported, together with the most recurrent genotype patterns. Frameshift variants at position Val142 and truncating mutations such as *Trp371X* and the exon-4 duplication (*c.409_424dup16*) were among the most frequently observed across symptoms. Wolframin Class 0—corresponding to predicted absence of functional protein—was predominant in patients with most neurological features, reflecting the substantial impact of severe protein-loss mutations on the multisystemic phenotype of Wolfram syndrome.

Symptom	Onset (yrs)	M (%)	Homozygotes (%)	Key Genetic Findings	Wolframin Class
Dysphagia	23.1 ± 7.3	66.7	63	Val142fsX110.	Type 0 (>70%)
Sialorrhea	24.0 ± 7.4	60.0	68	Val142fsX110.	Type 0 (68%)
Absence of Gag Reflex	23.4 ± 6.4	53.3	63	Val142fsX110 & Trp371X.	Type 0 (67%)
Dysmetria	24.1 ± 5.7	60.0	75	Val142fsX110.	Type 0 (80%)
Gait Instability	26.0 ± 4.5	62.5	62	Val142fsX110 & ex4 c.409_424dup16.	Type 0 (69%)
Ataxia	26.0 ± 4.5	62.5	75	Val142fsX110.	Type 0 (83%)
Cognitive Decline	29.9 ± 12.2	56.2	81	Val142fsX110 & Trp371X.	Type 0 (81%)
Anosmia	26.3 ± 6.2	55.6	78	Val142fs251 &Val142fsX110.	Type 0 (78%)
Impaired Tandem Gait	21.8 ± 6.0	62.1	66	Val142fs*, ex4c.409_424dup16.	Type 0 (69%)
Dysarthria	27.5 ± 8.1	53.9	77	Val142fs*, ex8 c.2206 G>A.	Type 0 (69%)
Adiadochokinesia	26.3 ± 8.3	58.9	82	Val142fs*,ex4 c.409_424dup16.	Type 0 (76%)

X denotes a stop codon. In frameshift variants (fs), the asterisks are part of the HGVS protein-variant nomenclature (e.g., fs*29 indicates a frameshift with a premature stop codon 29 amino acids downstream).

**Table 8:** Performance of genotype-based prediction models for each neurological symptom. Mean classification performance is reported for all neurological outcomes using repeated stratified cross-validation with 20 repetitions and 5 folds (100 resamples). Metrics include mean AUC and its standard deviation, accuracy, precision, recall, and F1-score. Confusion matrices correspond to aggregated predictions pooled across all folds and repetitions, providing a robust estimate of out-of-sample generalization for each symptom-specific machine learning model.

Symptom	Prevalence	Train Accuracy	Accuracy	AUC	Precision	Recall	F1-score	Confusion Matrix
Dysphagia	60.00%	0.813	0.578 ± 0.134	0.642 ± 0.156	0.634 ± 0.120	0.697 ± 0.218	0.649 ± 0.148	$\left(\begin{array}{cc} 145 & 215\\ 165 & 375 \end{array}\right)$
Sialorrhea	55.60%	0.827	0.653 ± 0.118	0.682 ± 0.169	0.681 ± 0.142	0.742 ± 0.199	0.694 ± 0.134	$\left(\begin{array}{cc} 217 & 183\\ 129 & 371 \end{array}\right)$
Absent_gag_reflex	66.70%	0.883	0.659 ± 0.147	0.691 ± 0.184	0.755 ± 0.121	0.742 ± 0.193	0.733 ± 0.138	$\left(\begin{array}{cc} 148 & 152\\ 155 & 445 \end{array}\right)$
Dysmetria	44.40%	0.796	0.643 ± 0.134	0.711 ± 0.168	0.607 ± 0.176	0.667 ± 0.229	0.614 ± 0.158	$\left(\begin{array}{cc} 312 & 188\\ 133 & 267 \end{array}\right)$
Gait_instability	64.40%	0.848	0.727 ± 0.131	0.754 ± 0.171	0.803 ± 0.120	0.787 ± 0.178	0.781 ± 0.117	$\left(\begin{array}{cc} 199 & 121\\ 125 & 455 \end{array}\right)$
Ataxia	53.30%	0.875	0.743 ± 0.116	0.839 ± 0.127	0.766 ± 0.157	0.797 ± 0.164	0.765 ± 0.114	$\left(\begin{array}{cc} 287 & 133\\ 98 & 382 \end{array}\right)$
Cognitive_impairment	35.60%	0.775	0.666 ± 0.141	0.725 ± 0.155	0.546 ± 0.191	0.736 ± 0.237	0.604 ± 0.169	$\left(\begin{array}{cc} 363 & 217\\ 84 & 236 \end{array}\right)$
Anosmia	40.00%	0.799	0.649 ± 0.139	0.732 ± 0.155	0.579 ± 0.215	0.618 ± 0.236	0.571 ± 0.183	$\left(\begin{array}{cc} 363 & 177\\ 139 & 221 \end{array}\right)$
Tandem_gait_abnormal	64.40%	0.802	0.647 ± 0.158	0.645 ± 0.183	0.745 ± 0.153	0.704 ± 0.211	0.706 ± 0.156	$\left(\begin{array}{cc} 174 & 146\\ 172 & 408 \end{array}\right)$
Dysarthria	28.90%	0.738	0.571 ± 0.147	0.606 ± 0.170	0.354 ± 0.223	0.575 ± 0.329	0.412 ± 0.221	$\left(\begin{array}{cc} 366 & 274\\ 112 & 148 \end{array}\right)$
Adiadochokinesia	37.80%	0.781	0.730 ± 0.134	0.720 ± 0.192	0.622 ± 0.175	0.802 ± 0.223	0.684 ± 0.166	$\left(\begin{array}{cc} 383 & 177\\ 66 & 274 \end{array}\right)$

**Table 9:** Summary of calibration results for the neurological symptoms associated with Wolfram syndrome. Raw Random Forest probabilities were systematically miss-calibrated, but both isotonic and Platt calibration markedly improved discrimination (AUC) and probability accuracy (Brier score) across all symptoms. Symptoms with strong cerebellar or brainstem involvement showed the highest calibrated performance, while more heterogeneous symptoms showed lower predictability even after calibration.

Symptom	AUC-R	AUC-I	AUC-P	Brier -R	Brier-I	Brier-P
Dysphagia	0.550	0.888	0.871	0.290	0.146	0.216
Sialorrhea	0.595	0.934	0.938	0.249	0.124	0.17
Absent gag reflex	0.647	0.971	0.962	0.195	0.075	0.126
Dysmetria	0.627	0.916	0.934	0.280	0.117	0.186
Gait instability	0.690	0.961	0.971	0.221	0.079	0.14
Ataxia	0.823	0.964	0.968	0.178	0.059	0.108
Cognitive impairment	0.656	0.927	0.939	0.250	0.115	0.154
Anosmia	0.643	0.907	0.919	0.272	0.127	0.169
Impaired tandem gait	0.575	0.940	0.873	0.296	0.122	0.178
Dysarthria	0.589	0.921	0.916	0.261	0.124	0.192
Adiadochokinesia	0.653	0.918	0.922	0.244	0.134	0.171

**Table 10:** Cross-diagnostic summary of dominant, secondary, and marginal predictors across neurological phenotypes in Wolfram syndrome. This table synthesizes the relative importance of genetic and interaction features across all symptom-specific models. Dominant predictors refer to attributes that consistently showed the strongest global influence within each model, typically reflecting the direct effect of protein-altering mutations on alleles 1 and 2. Secondary predictors represent interaction effects—most frequently combinations of mutation class, exon context, and genetic condition—that contributed meaningfully but with lower magnitude. Weak or marginal predictors include wolframin class and exon-level features that exhibited only limited or inconsistent impact across disorders. Together, these patterns highlight the predominance of protein-level mutation effects, supplemented by moderate contributions from genetic interaction terms, in shaping the heterogeneous neurological phenotype of WFS1-related disease.

Symptom	Dominant Predictors	Consistent Secondary Predictors	Weak/Marginal Predictors
Dysphagia	mut1_protein, mut2_protein	prod_mgm1/mgm2/mgm12.	Wolframin features, exon classes
Symptom	Dominant Predictors	Consistent Secondary Predictors	Weak/Marginal Predictors
Sialorrhea	mut2_protein, mut1_protein	prod_mgm1/mgm12/mgm2; mut12_exon_class	Wolframin features
Absent Gag Reflex	mut1_protein, mut2_protein	prod_mgm1/mgm2/mgm12, mut12_exon_class	Wolframin features, genomic context
Dysmetria	mut2_protein, mut1_protein	prod_mgm1/mgm2; Wolframin features	Exon features
Gait Instability	mut2_protein, mut1_protein	prod_mgm1/mgm2; prod_wm1/wm2/wm12; wolframin_class; Genetic_Condition	Exon-level variables; prod_wmg; mut12_exon_class
Ataxia	mut2_protein, mut1_protein	prod_mgm1/mgm2	Wolframin features, exon classes, genomic context (weak)
Cognitive Impairment	mut1_protein, mut2_protein	prod_mgm1/mgm2; Genetic_Condition	Wolframin features, exon classes
Anosmia	mut1_protein, mut2_protein	prod_mgm2; small WM contributions	Exon classes, prod features
Tandem Gait Impairment	mut2_protein, mut1_protein	prod_mgm1/mgm2/mgm12	Wolframin features, exon classes (very small)
Dysarthria	mut1_protein, mut2_protein	prod_mgm1/mgm2/mgm12	Wolframin features, exon classes (small)
Adiadochokinesia	mut1_protein, mut2_protein	prod_mgm1/mgm12/mgm2	Wolframin features

## Data Availability

The data presented in this study are available on request from the corresponding author. The data are not publicly available due to patient privacy and ethical restrictions related to rare disease research.

## Supplementary Materials

The following supporting information can be downloaded at: https://www.mdpi.com/article/10.3390/diagnostics15243213/s1, Table S1. Genotype–phenotype correlations in the Spanish Wolfram syndrome cohort; Table S2. Dysphagia—Relevant Predictors; Table S3. Sialorrhea—Relevant Predictors; Table S4. Absent Gag Reflex—Relevant Predictors; Table S5. Dysmetria—Relevant Predictors; Table S6. Gait Instability—Relevant Predictors; Table S7. Ataxia—Relevant Predictors; Table S8. Cognitive Impairment—Relevant Predictors; Table S9. Anosmia—Relevant Predictors; Table S10. Tandem Gait Impairment—Relevant Predictors; Table S11. Dysarthria—Relevant Predictors; Table S12. Adiadochokinesia—Relevant Predictors; Figure S1. Cross-validated ROC curves for each neurological disorder.

## References

[1] Barrett T.G., Bundey S.E., Macleod A.F. (1995). Neurodegeneration and diabetes: UK nationwide study of Wolfram (DIDMOAD) syndrome. Lancet.

[2] Cremers C., Wijdeveld P., Pinckers A. (1977). Juvenile diabetes mellitus, optic atrophy, hearing loss, diabetes insipidus, atonia of the urinary tract and bladder, and other abnormalities (Wolfram syndrome). A review of 88 cases from the literature with personal observations on 3 new patients. Acta Paediatr. Scand. Suppl..

[3] Urano F. (2016). Wolfram syndrome: Diagnosis, management, and treatment. Curr. Diabetes Rep..

[4] Inoue H., Tanizawa Y., Wasson J., Behn P., Kalidas K., Bernal-Mizrachi E., Mueckler M., Marshall H., Donis-Keller H., Crock P. (1998). A gene encoding a transmembrane protein is mutated in patients with diabetes mellitus and optic atrophy (Wolfram syndrome). Nat. Genet..

[5] Fonseca S.G., Fukuma M., Lipson K.L., Nguyen L.X., Allen J.R., Oka Y., Urano F. (2005). WFS1 is a novel component of the unfolded protein response and maintains homeostasis of the endoplasmic reticulum in pancreatic β cells. J. Biol. Chem..

[6] Lee E.M., Verma M., Palaniappan N., Pope E.M., Lee S., Blacher L., Neerumalla P., An W., Campbell T., Brown C. (2023). Genotype and clinical characteristics of patients with Wolfram syndrome and WFS1-related disorders. Front. Genet..

[7] Antenora A., Lieto M., Santorelli F.M., Peluso S., Saccà F., De Michele G., Filla A. (2016). Be aware of Wolfram syndrome when examining ataxic patients. J. Neurol..

[8] Samara A., Lugar H.M., Hershey T., Shimony J.S. (2020). Longitudinal assessment of neuroradiologic features in Wolfram syndrome. AJNR Am. J. Neuroradiol..

[9] Lugar H.M., Koller J.M., Rutlin J., Eisenstein S.A., Neyman O., Narayanan A., Chen L., Shimony J.S., Hershey T. (2019). Evidence for altered neurodevelopment and neurodegeneration in Wolfram syndrome using longitudinal morphometry. Sci. Rep..

[10] Hershey T., Lugar H.M., Shimony J.S., Rutlin J., Koller J.M., Perantie D.C., Paciorkowski A.R., Eisenstein S.A., Permutt M.A. (2012). Early brain vulnerability in Wolfram syndrome. PLoS ONE.

[11] Esteban-Bueno G., Fernández-Martínez J.L. (2025). Gonadal dysfunction in Wolfram syndrome: A prospective study. Diagnostics.

[12] Esteban-Bueno G., Berenguel Hernández A.M., Fernández Fernández N., Navarro Cabrero M., Coca J.R. (2023). Neurosensory affectation in patients affected by Wolfram syndrome: Descriptive and longitudinal analysis. Healthcare.

[13] Chen C., Liaw A., Breiman L. (2004). Using Random Forest to Learn Imbalanced Data.

[14] Pickett K.A., Duncan R.P., Hoekel J., Marshall B., Hershey T., Earhart G.M. (2012). Early presentation of gait impairment in Wolfram Syndrome. Orphanet J. Rare Dis..

[15] Atiq E.S.A., Ghatak A., Raj A. (2024). 41 A case of wolfram syndrome: Understanding a rare genetic disorder. BMJ Paediatr. Open.

[16] Lemaître G., Nogueira F., Aridas C.K. (2017). Imbalanced-learn: A Python toolbox to tackle the curse of imbalanced datasets in machine learning. J. Mach. Learn. Res..

[17] Zadrozny B., Elkan C. Transforming classifier scores into accurate multiclass probability estimates. Proceedings of the Eight ACM SIGKDD International Conference on Knowledge Discovery and Data Mining.

[18] Platt J.C., Smola A.J., Bartlett P.L., Schölkopf B., Schuurmans D. (1999). Probabilistic outputs for support vector machines and comparisons to regularized likelihood methods. Advances in Large Margin Classifiers.

[19] Brier G.W. (1950). Verification of forecasts expressed in terms of probability. Mon. Weather Rev..

[20] Breiman L. (2001). Random forests. Mach. Learn..

[21] Kuhn M., Johnson K. (2013). Applied Predictive Modeling.

[22] Jurca A.D., Galea-Holhos L.B., Jurca A.A., Atasie D., Petchesi C.D., Severin E., Jurca C.M. (2024). Wolfram syndrome type I case report and review—Focus on early diagnosis and genetic variants. Medicina.

[23] Du D., Tuhuti A., Ma Y., Abuduniyimu M., Li S., Ma G., Zynat J., Guo Y. (2023). Wolfram syndrome type 1: A case series. Orphanet J. Rare Dis..

[24] Delvecchio M., Iacoviello M., Pantaleo A., Resta N. (2021). Clinical spectrum associated with Wolfram syndrome type 1 and type 2: A review on genotype–phenotype correlations. Int. J. Environ. Res. Public Health.

[25] Rigoli L., Caruso V., Salzano G., Lombardo F. (2022). Wolfram syndrome 1: From genetics to therapy. Int. J. Environ. Res. Public Health.

[26] Gupta P., Vu T.A., Lamoureux E.L. (2021). Beyond Visual Acuity—A Comprehensive Assessment of Vision and Cognition in Older Adults With Visual Impairment. JAMA Netw. Open.

[27] Lin F.R., Ferrucci L., Metter E.J., An Y., Zonderman A.B., Resnick S.M. (2011). Hearing loss and cognition in the Baltimore Longitudinal Study of Aging. Neuropsychology.

[28] Taffner B., Saraiva P., Saraiva F. (2015). Wolfram Syndrome: Report of Three Siblings with Early Vision Loss and Documented Lesion of the Entire Visual Pathway. Ophthalmol. Res. Int. J..

[29] Reiersen A.M., Noel J.S., Doty T., Sinkre R.A., Narayanan A., Hershey T. (2022). Psychiatric diagnoses and medications in Wolfram syndrome. Scand. J. Child Adolesc. Psychiatry Psychol..

[30] Warren R.E., Frier B.M. (2005). Hypoglycaemia and cognitive function. Diabetes Obes. Metab..

[31] Fonseca L.M., Strong R.W., Singh S., Bulger J.D., Cleveland M., Grinspoon E., Janess K., Jung L., Miller K., Passell E. (2023). Glycemic Variability and Fluctuations in Cognitive Status in Adults With Type 1 Diabetes (GluCog): Observational Study Using Ecological Momentary Assessment of Cognition. JMIR Diabetes.

[32] Pickett K.A., Duncan R.P., Paciorkowski A.R., Permutt M.A., Marshall B., Hershey T., Earhart G.M. (2012). Balance impairment in individuals with Wolfram syndrome. Gait Posture.

[33] U J., Santhanam J., Rm R., Saideekshit T., Sn M.S. (2024). Beyond Vision and Hearing: A Case Report of Wolfram Syndrome. Cureus.

[34] Meng X., D’Arcy C. (2012). Education and dementia in the context of the cognitive reserve hypothesis: A systematic review with meta-analyses and qualitative analyses. PLoS ONE.

[35] Mondini S., Madella I., Zangrossi A., Bigolin A., Tomasi C., Michieletto M., Villani D., Di Giovanni G., Mapelli D. (2016). Cognitive reserve in dementia: Implications for cognitive training. Front. Aging Neurosci..

[36] Caruso V., Raia A., Rigoli L. (2024). Wolfram Syndrome 1: A Neuropsychiatric Perspective on a Rare Disease. Genes.

[37] de Azevedo M.C.D., Charchat-Fichman H., Damazio V.M.M. (2021). Environmental interventions to support orientation and social engagement of people with Alzheimer’s disease. Dement. Neuropsychol..

[38] Mendes L., Oliveira J., Barbosa F., Castelo-Branco M. (2022). A Conceptual View of Cognitive Intervention in Older Adults With and Without Cognitive Decline—A Systemic Review. Front. Aging.

[39] Esteban-Bueno G., Jiménez-Soto A., Fernández-Martínez J.L., Fernández-Vilas E., Coca J.R. (2025). Circadian Rhythm and Psychiatric Features in Wolfram Syndrome: Toward Chrono Diagnosis and Chronotherapy. Diagnostics.

