# Supplementary material for: Phenotype Correlations of Neurological Manifestations in Wolfram Syndrome: Predictive Modeling in a Spanish Cohort
Source: Diagnostics (Basel). 2025 Dec 16;15(24):3213. doi: 10.3390/diagnostics15243213 (PMC12732145; doi:10.3390/diagnostics15243213)
Supplement: Supplementary file 1 [file diagnostics-15-03213-s001.zip › diagnostics-3980686-supplementary.pdf]

Supplementary Material

Table S1. Genotype–phenotype correlations in the Spanish Wolfram syndrome cohort. The table lists all genotype–phenotype pairs with an absolute correlation coefficient ≥ 0.400. Neurological conditions are shown together with the corresponding genetic variables and their correlation values (rounded to three decimals). These results highlight the strongest linear associations between specific WFS1 genetic features and neurological manifestations, supporting the presence of partially conserved genotype–phenotype patterns within the cohort.

Neurological Condition	Genetic Variable	Correlation (3 decimals)
Ataxia	prod_wmg	0.567
Ataxia	prod_wm12	0.561
Ataxia	prod_mgm12	0.555
Ataxia	mut12_exon_class	0.552
Ataxia	wolframin_class	0.544
Ataxia	prod_mgm2	0.541
Ataxia	prod_wm1	0.521
Ataxia	prod_wm2	0.521
Ataxia	genetic_condition	0.511
Adiadochokinesia	prod_mgm2	0.510
Adiadochokinesia	genetic_condition	0.487
Sialorrhea	mut12_exon_class	0.486
Adiadochokinesia	prod_mgm12	0.476
Cognitive impairment	prod_mgm2	0.469
Sialorrhea	prod_mgm12	0.467
Adiadochokinesia	prod_mgm1	0.466
Anosmia	prod_mgm2	0.459
Dysmetria	prod_mgm2	0.452
Ataxia	prod_mgm1	0.449
Cognitive impairment	genetic_condition	0.448
Anosmia	prod_mgm12	0.447
Dysmetria	prod_wmg	0.443
Cognitive impairment	prod_mgm12	0.440

Anosmia	mut12_exon_class	0.439
Anosmia	genetic_condition	0.436
Cognitive impairment	prod_mgm1	0.429
Dysmetria	genetic_condition	0.427
Cognitive impairment	prod_wmg	0.427
Dysmetria	prod_mgm1	0.424
Gait instability	prod_wm1	0.424
Gait instability	prod_wm2	0.424
Impaired tandem gait	prod_mgm2	0.423
Ataxia	type_mut2_exon	0.422
Dysmetria	prod_mgm12	0.416
Sialorrhea	prod_mgm2	0.407
Anosmia	prod_mgm1	0.404
Dysphagia	prod_wm12	0.403

Cross-Phenotype Determinants of Clinical Manifestations in WFS1 Spectrum Disorders

Table S2. Dysphagia — Relevant Predictors.

In dysphagia, mut1_protein_class and mut2_protein_class show the strongest effects, with high stability and large full-model permutation values. Mut2_protein_class shows the largest full-model importance, while mut1_protein_class displays a substantial cross-validated effect, indicating that protein-level pathogenicity on both alleles is the central determinant of dysphagia, with allele-2 effects exerting slightly greater influence. Interaction terms contribute only weakly. prod_mgm2 appears consistently across importance metrics with high stability but very small effect sizes, suggesting a reproducible yet minimal contribution. prod_mgm1 and prod_mgm12 follow with similarly small, stable signals. These interaction terms remain detectable but do not approach the magnitude of the protein-class predictors. Lower-ranked features—including prod_wm1, prod_wm2, wolframin_class, and Genetic_Condition_class—show minimal or marginal contribution, with low stability and small or inconsistent permutation effects. Exome-level features such as mut12_exom_class and mut2_exom_class also display weak signals, indicating limited discriminative value once the dominant protein-class predictors are included. Dysphagia is primarily driven by the severity of the mutation's impact at the protein level, with only minor additional influence from interaction terms or broader genetic-context variables.

Feature	Stability	CV Perm ± SD	CV Gini	Full Perm ± SD	Full Gini	Interpretation
mut1_protein_class	0.96	0.029 ± 0.034	0.203	0.079 ± 0.032	0.239	Strong genetic driver; robust across datasets.
mut2_protein_class	0.97	0.008 ± 0.029	0.204	0.107 ± 0.037	0.296	Strongest full-model predictor; consistent allele-2 dominance.
prod_mgm1	0.43	0.004 ± 0.021	0.090	0.004 ± 0.009	0.036	Weak but consistent across models.
prod_mgm2	0.93	−0.005 ± 0.024	0.099	0.003 ± 0.008	0.039	Stable but effect small; likely downstream noise.
prod_mgm12	0.50	−0.010 ± 0.024	0.090	0.006 ± 0.011	0.047	Weak but present in both models.
prod_wm1	0.55	0.002 ± 0.015	0.034	−0.003 ± 0.013	0.041	Very small but non-random signal.
prod_wm2	0.50	−0.003 ± 0.013	0.035	0.003 ± 0.012	0.052	Low contribution; could reflect WM pathway effects.
mut12_exon_class	0.60	−0.005 ± 0.014	0.051	0.020 ± 0.012	0.052	Small genetic secondary effect.
type_mut2_exon	0.45	0.005 ± 0.011	0.028	−0.002 ± 0.013	0.027	Weak, likely noise.
wolframin_class	0.40	0.000 ± 0.013	0.028	0.002 ± 0.010	0.043	Minimal predictive value.
genetic_condition	0.30	−0.004 ± 0.021	0.064	0.004 ± 0.010	0.018	Very small, marginal effect.

Table S3. Sialorrhea — Relevant Predictors.

In sialorrhea, mut2_protein_class is the strongest and most stable predictor, with consistently high importance across cross-validated permutation, full-model permutation, and Gini metrics. mut1_protein_class follows as a secondary but still substantial contributor, reflecting a clear protein-level dominance in the predictive structure. A set of interaction terms provides additional reproducible signal. prod_mgm12 shows very high stability and consistent importance across all measures, indicating a robust secondary contribution. prod_mgm1 and prod_mgm2 show moderate and stable effects, although with more variability across folds, suggesting meaningful but less dominant interaction influences. mut12_exom_class appears as a small but clearly present predictor, with consistent effect sizes in both cross-validated and full-model analyses. Lower-ranked features, including prod_wm1, prod_wm2, wolframin_class, and Genetic_Condition_class, show minimal or negligible contribution, with weak and unstable effects and very low importance across all metrics. These predictors add little discriminative value once the main protein-level and key interaction terms have been accounted for. Sialorrhea is primarily driven by protein-level effects, with additional moderate influence from specific interaction terms and only minimal contributions from broader genetic context variables.

**Feature**	**Stability**	**CV Perm ± SD**	**CV Gini**	**Full Perm ± SD**	**Full Gini**	**Interpretation**
mut2_protein_class	1.00	0.028 ± 0.029	0.204	0.113 ± 0.039	0.263	Strongest and most stable predictor.
mut1_protein_class	0.78	0.004 ± 0.035	0.203	0.047 ± 0.023	0.215	Secondary but strong protein-level effect.
prod_mgm1	0.61	0.019 ± 0.020	0.090	0.004 ± 0.008	0.038	Moderate but somewhat unstable.
prod_mgm12	0.96	0.010 ± 0.023	0.090	0.010 ± 0.011	0.088	Consistent secondary contributor.
prod_mgm2	0.70	0.008 ± 0.023	0.099	0.004 ± 0.012	0.055	Weak but stable across datasets.
mut12_exon_class	0.89	0.015 ± 0.013	0.051	0.016 ± 0.011	0.084	Small but biologically relevant.
prod_wm1	0.40	0.009 ± 0.014	0.034	−0.003 ± 0.010	0.033	Minimal signal.
prod_wm2	0.30	0.003 ± 0.013	0.035	−0.004 ± 0.010	0.030	Very small effect.
wolframin_class	0.35	0.006 ± 0.013	0.028	−0.004 ± 0.010	0.031	Weak.
genetic_condition	0.25	0.003 ± 0.021	0.064	0.005 ± 0.008	0.030	Negligible.

Table S4. Absent Gag Reflex— Relevant Predictors**.**

In absent gag reflex, mut1_protein_class shows the strongest and most stable effect across cross-validated and full-model metrics, indicating that protein-level severity on allele 1 contributes substantially to the impairment. mut2_protein_class also displays a strong influence, particularly in the full-model permutation and Gini importance, although its cross-validated permutation effect is smaller, reflecting greater variability across folds. The interaction terms prod_mgm1 and prod_mgm2 form a secondary cluster of predictors with moderate and consistent contributions. Their stability and reproducibility across importance measures indicate that these allele-configuration interactions add meaningful information beyond the protein-class predictors. mut12_exom_class and prod_mgm12 appear as smaller yet still present contributors, with effect sizes that remain detectable across both CV and full-model analyses. Several additional interaction terms, including prod_wm2 and prod_wm1, show weak but non-random influence, with small, reproducible effects that remain visible across importance metrics. Features such as wolframin_class, Genetic_Condition_class, and prod_wmg fall at the lower end of the ranking, with minimal signal and limited stability, indicating that they add little discriminative value once the principal protein-level and interaction terms are included. Absent gag reflex is shaped primarily by protein-level effects on both alleles, with moderate contributions from key interaction terms and only minimal influence from broader genomic context or higher-order interactions.

Feature	Stability	CV Perm ± SD	CV Gini	Full Perm ± SD	Full Gini	Interpretation
mut1_protein_class	0.90	0.007 ± 0.029	0.203	0.051 ± 0.023	0.210	Strong, stable predictor for reflex impairment.
mut2_protein_class	0.75	0.004 ± 0.027	0.204	0.084 ± 0.031	0.263	Strong full-model effect; CV weaker due to noise.
prod_mgm1	0.72	0.005 ± 0.021	0.090	0.013 ± 0.007	0.060	Moderate consistent contribution.
prod_mgm2	0.62	−0.005 ± 0.025	0.099	0.011 ± 0.010	0.062	Weak but stable; meaningful secondary effect.
mut12_exon_class	0.55	−0.004 ± 0.013	0.051	0.012 ± 0.011	0.056	Secondary genetic influence.
prod_mgm12	0.50	−0.006 ± 0.025	0.090	0.008 ± 0.007	0.055	Small but consistent.
prod_wm2	0.50	−0.004 ± 0.014	0.035	0.005 ± 0.009	0.050	Weak but present.
prod_wm1	0.45	−0.008 ± 0.014	0.034	0.006 ± 0.008	0.043	Very small effect.
wolframin_class	0.30	−0.000 ± 0.013	0.028	0.002 ± 0.009	0.031	Minimal contribution.
genetic_condition	0.20	−0.014 ± 0.022	0.064	0.005 ± 0.006	0.029	Negligible.
prod_wmg	0.15	−0.004 ± 0.012	0.019	0.004 ± 0.007	0.015	Tiny effect.

Table S5. Dysmetria — Relevant Predictors.

In dismetria, mut2_protein_class and mut1_protein_class emerge as the dominant and fully stable predictors, with both showing perfect stability (1.00) and large, consistent effects across cross-validated permutation, Gini importance, and full-model permutation. Mut2 exhibits the strongest overall signal, while Mut1 follows closely, indicating that protein-level pathogenicity on each allele independently explains most of the variance in dismetria. The strength and reproducibility of these two predictors highlight a tightly protein-driven phenotype. A coherent secondary predictor group is formed by prod_mgm1 and prod_mgm2, each of which displays moderate importance and strong consistency across metrics. Their stability suggests that combinatorial allele-level interactions captured by these terms provide meaningful additional predictive information, complementing (but not rivaling) the dominant protein-class effects. Several other interaction terms—including prod_wm1, prod_wm2, mut12_exom_class, and prod_mgm12—show small but non-random effects, with reproducible though modest contributions. These features appear consistently in the rankings with low magnitude, indicating subtle secondary influences that refine the model without driving its core structure. Lower-group predictors such as wolframin_class, prod_wm12, and Genetic_Condition_class exhibit very small, unstable, or background-level effects. Their low importance and variability suggest that once protein-class severity and the main interaction terms are accounted for, broad genetic context and higher-order interactions contribute only minimally to predicting dismetria. The model shows a strongly protein-centric predictive architecture, with dismetria primarily explained by the severity of protein-level impairment on both alleles, supported by moderate contributions from key interaction terms and only weak influence from broader genomic or exon-related variables. Dismetria is driven mainly by protein-level effects on both alleles, supported by moderate contributions from prod_mgm1 and prod_mgm2, with only small, secondary signals from interaction and exon-related predictors.

Feature	Stability	CV Perm ± SD	CV Gini	Full Perm ± SD	Full Gini	Interpretation
mut2_protein_class	1.00	0.064 ± 0.026	0.204	0.082 ± 0.027	0.254	Strongest and most stable predictor; consistent across models.
mut1_protein_class	1.00	0.051 ± 0.032	0.203	0.067 ± 0.023	0.227	Strong protein-level genetic effect, nearly as predictive as mut2.
prod_mgm1	0.78	0.014 ± 0.014	0.090	0.008 ± 0.012	0.053	Moderate contributor; consistent secondary effect.
prod_mgm2	0.70	0.003 ± 0.017	0.099	0.011 ± 0.012	0.060	Moderately important; stable across datasets.
prod_wm1	0.65	0.016 ± 0.014	0.034	−0.004 ± 0.013	0.056	Weak but not noise; appears consistently as a small effect.
prod_wm2	0.60	0.013 ± 0.013	0.035	−0.005 ± 0.013	0.057	Consistent minor effect; likely reflecting downstream impact.
mut12_exon_class	0.55	0.006 ± 0.015	0.051	0.015 ± 0.014	0.031	Small but real secondary genetic signal.
prod_mgm12	0.50	−0.001 ± 0.019	0.090	0.004 ± 0.013	0.046	Weak but present in both models.
wolframin_class	0.40	0.012 ± 0.013	0.028	−0.005 ± 0.012	0.046	Very small effect; likely background genetic context.
prod_wm12	0.35	0.008 ± 0.013	0.031	−0.004 ± 0.012	0.045	Minor and unstable.

Feature	Stability	CV Perm ± SD	CV Gini	Full Perm ± SD	Full Gini	Interpretation
genetic_condition	0.30	0.002 ± 0.017	0.064	0.007 ± 0.010	0.036	Weak genetic modifier.

Table S6. Gait Instability— Relevant Predictors. The feature-importance profile for this model is characterized by a dominant protein-level genetic axis, led by *mut2_protein_class* and *mut1_protein_class*, both of which show perfect stability (1.00) and consistently elevated importance across cross-validation permutation scores, full-model permutation values, and Gini metrics. Mut2 displays the strongest overall contribution, particularly in the full-model measures, while mut1 provides a slightly smaller but equally reproducible effect. Together, these features indicate that allele-specific protein consequences represent the primary determinants of model performance, with allele 2 exerting marginally greater influence. A second tier of predictors—including *wolframin_class*, *prod_wm1*, *prod_mgm1*, *prod_wm2*, and *prod_wm12*—shows moderate but stable contributions, reflected by coherent Gini rankings and small-to-moderate permutation effects. These features capture broader functional distinctions within wolframin and specific allele–protein interaction patterns, adding meaningful nuance to the strong primary protein-level signal. Their behavior suggests that structural and biochemical relationships between variants contribute additional explanatory information, even if their effect sizes remain secondary. A third group, consisting of *prod_mgm2*, *Type_mut2_exon*, *Genetic_Condition*, and *prod_wmg*, demonstrates low-to-modest influence, with small permutation scores and variable Gini metrics. These features appear to reflect zygosity-dependent modifiers and exome-level distinctions, providing incremental refinement rather than driving prediction. Their reproducibility across metrics indicates that they are not random, but their contributions remain overshadowed by dominant protein-level effects. Finally, lower-ranked features such as *prod_mgm12*, *Type_mut1_exon*, and *mut12_exon_class* exhibit minimal or unstable importance, with low stability and weak permutation signals. These predictors introduce negligible additional information once the major protein and interaction features are accounted for, and likely represent fine-grained genomic variation with limited predictive impact in this setting. The model exhibits a protein-centric predictive architecture, dominated by allele-specific protein-class effects and complemented by moderate contributions from interaction-based and wolframin-class features. Exome-level and higher-order interaction variables offer only small, context-dependent refinements. This structure indicates that the functional consequences of WFS1 mutations at the protein level form the core predictive signal, with secondary modulation arising from structural, metabolic, and zygosity-driven interactions.

Feature	Stability	CV Perm (mean ± SD)	CV Gini	Full Perm (mean ± SD)	Full Gini	Interpretation
mut2_protein_class	1.00	0.028 ± 0.021	0.0817	0.076 ± 0.026	0.2401	Strongest and most consistent predictor; dominant signal in both CV and Full models.
mut1_protein_class	1.00	0.013 ± 0.013	0.0656	0.028 ± 0.013	0.1728	Second strongest genetic feature; stable and reproducible contribution.
wolframin_class	0.70	0.006 ± 0.015	0.1144	0.005 ± 0.008	0.0396	Moderate CV Gini signal; weaker and less consistent in Full model.
prod_wm1	0.65	0.005 ± 0.023	0.2026	0.007 ± 0.012	0.0932	High CV Gini but weak permutation signal; secondary structural contributor.
prod_mgm1	0.85	0.010 ± 0.014	0.0512	0.008 ± 0.008	0.0530	Stable mild-to-moderate effect; consistently selected across runs.

Feature	Stability	CV Perm (mean ± SD)	CV Gini	Full Perm (mean ± SD)	Full Gini	Interpretation
prod_wm2	0.80	0.004 ± 0.025	0.2317	0.007 ± 0.012	0.1075	Strong CV Gini but small perm values; likely reflecting a subtle but real WM effect.
prod_wm12	0.75	0.004 ± 0.016	0.1127	0.007 ± 0.008	0.0509	Recurrent but modest contributor; complementary to wm1 and wm2.
prod_mgm2	0.80	0.007 ± 0.013	0.0257	0.009 ± 0.007	0.0414	Secondary metabolic marker with mild but consistent importance.
type_mu2_exon	0.60	0.016 ± 0.008	0.0061	0.001 ± 0.006	0.0305	CV permutation signal present, but largely disappears in Full model.
genetic_condition	0.70	0.007 ± 0.012	0.0147	0.008 ± 0.007	0.0259	Low-level global genetic effect; consistent but non-dominant.
prod_wmg	0.65	0.004 ± 0.013	0.0320	0.008 ± 0.006	0.0224	Minor contributor; weak but repeatedly observed.
prod_mgm12	0.60	0.004 ± 0.012	0.0176	0.007 ± 0.008	0.0410	Very small effect; may capture composite metabolic interactions.
type_mut1_exon	0.60	0.003 ± 0.011	0.0311	0.003 ± 0.007	0.0396	Weak predictor; exome-level resolution adds limited information.
mut12_exon_class	0.50	0.001 ± 0.010	0.0128	0.009 ± 0.011	0.0422	Minimal CV effect but mild Full-model signal; low stability.

Table S7. Ataxia — Relevant Predictors.

In ataxia, mut2_protein_class and mut1_protein_class clearly dominate the predictive landscape, with both features showing perfect stability (1.00) and consistently high importance across all metrics. Mut2 displays the strongest overall effect—especially in the full-model permutation and Gini scores—while mut1 provides a slightly smaller but equally robust contribution. Together, these results indicate that protein-level pathogenicity on both alleles is the primary determinant of ataxia, with allele 2 contributing marginally more predictive power. A second group of predictors is formed by the prod_mgm1 and prod_mgm2 interaction terms. These show moderate and stable contributions across cross-validation, Gini rankings, and full-model permutation, suggesting that allelic configuration interacting with mutation-specific characteristics provides meaningful additional information beyond the protein-class effects. The small but reproducible influence of Genetic_Condition_class reinforces this pattern, indicating that broader zygosity patterns modestly modulate ataxia risk. Further interaction terms such as prod_mgm12, mut2_exom_class, and wolframin_class show weak but non-random contributions, persisting across importance metrics but with lower effect sizes. Their behavior suggests subtle, secondary variations in risk that complement but do not compete with the dominant protein-class predictors. Lower-ranked features—including mut1_exom_class, prod_wm1, prod_wm2, mut12_exom_class, prod_wm12, and prod_wmg—show very small or unstable effects, with low stability and inconsistent directionality across permutation and Gini metrics. These predictors contribute minimal additional information once the dominant protein and main interaction terms are accounted for. The ataxia model presents a protein-centric predictive structure, strongly driven by allele-specific protein consequences, with moderate contributions from genetic-interaction terms and only minor influence from broader genomic or exon-based features. Ataxia is primarily driven by strong and stable protein-level effects on both alleles, with moderate contributions from key interaction terms and only weak, inconsistent influence from exome-level variables and higher-order interactions.

Feature	Stability	CV Perm ± SD	CV Gini	Full Perm ± SD	Full Gini	Interpretation
mut2_protein_class	1.00	0.037 ± 0.023	0.204	0.056 ± 0.023	0.213	Strongest and most stable predictor of ataxia.
mut1_protein_class	1.00	0.026 ± 0.028	0.203	0.022 ± 0.013	0.145	Strong secondary predictor; very stable.
prod_mgm1	0.80	0.018 ± 0.015	0.090	0.007 ± 0.007	0.050	Moderate, consistent effect across models.
prod_mgm2	0.75	0.007 ± 0.018	0.099	0.007 ± 0.008	0.062	Weak but stable functional predictor.
genetic_condition	0.70	0.006 ± 0.017	0.064	0.007 ± 0.007	0.031	Small but consistent signal reflecting broad genomic context.
prod_mgm12	0.60	0.004 ± 0.020	0.090	0.005 ± 0.009	0.060	Weak but present in both models.
type_mut2_exon	0.55	0.008 ± 0.009	0.028	0.001 ± 0.012	0.026	Minor secondary genetic contribution.
wolframin_class	0.55	0.007 ± 0.011	0.028	−0.001 ± 0.011	0.081	Weak and somewhat unstable signal.

Feature	Stability	CV Perm ± SD	CV Gini	Full Perm ± SD	Full Gini	Interpretation
type_mut1_exon	0.45	0.007 ± 0.008	0.022	−0.002 ± 0.008	0.015	Very small effect; low importance.
prod_wm1	0.45	0.006 ± 0.013	0.034	−0.001 ± 0.010	0.074	Weak white-matter related effect.
prod_wm2	0.40	0.004 ± 0.012	0.035	−0.001 ± 0.010	0.078	Very small effect.
mut12_exon_class	0.30	−0.003 ± 0.011	0.051	0.004 ± 0.008	0.048	Negligible influence.
prod_wm12	0.25	0.002 ± 0.011	0.031	−0.001 ± 0.010	0.077	Almost no signal.
prod_wmg	0.20	0.004 ± 0.011	0.019	0.001 ± 0.008	0.042	Minimal effect; likely noise.

Table S8. Cognitive Impairment — Relevant Predictors

In cognitive impairment, mut1_protein_class and mut2_protein_class stand out as the dominant and most stable predictors, confirming that the severity of the protein-level impact on both alleles is the principal driver of cognitive involvement. Mut2 shows the strongest full-model effect, while Mut1 exhibits the largest cross-validated permutation influence, indicating robust and complementary contributions from both alleles across modeling schemes. A coherent secondary predictor cluster is formed by prod_mgm1 and prod_mgm2, both of which display moderate, stable importance across cross-validation, Gini, and full-model permutation. This pattern shows that combinatorial allele-level interactions captured by these terms provide additional explanatory power, above and beyond the main protein-effect variables. The consistent but smaller signal of Genetic_Condition_class reinforces the idea that overall allelic configuration makes a measurable—though modest—contribution to cognitive impairment. Weak but reproducible effects are also observed for prod_mgm12, prod_wm1, prod_wm2, and prod_wmg, suggesting that higher-order interaction patterns contribute minimally yet non-randomly. These features persist across metrics, but their effect sizes remain small compared to the dominant protein-level classes. Exome-derived predictors show little to no stable influence: mut12_exom_class, mut2_exom_class, and mut1_exom_class exhibit low importance, inconsistent directionality (negative CV effects, small positive full-model effects), and low stability. Similarly, prod_wm12 displays negligible predictive value. The model reveals a protein-centric architecture for cognitive impairment, with moderate contributions from specific combinatorial interaction terms and only minor influence from broader genomic or exon-level variables once the main protein-class predictors are accounted for. Cognitive impairment is primarily driven by protein-level mutation severity, with moderate additional influence from prod_mgm1 and prod_mgm2, and only weak secondary effects from global genetic context and higher-order interaction terms.

Feature	Stability	CV Perm ± SD	CV Gini	Full Perm ± SD	Full Gini	Interpretation
mut1_protein_class	0.85	0.089 ± 0.032	0.203	0.050 ± 0.027	0.193	Strong genetic driver; very large CV effect.
mut2_protein_class	0.90	0.059 ± 0.028	0.204	0.095 ± 0.032	0.251	Strongest full-model predictor; highly stable.
prod_mgm1	0.80	0.019 ± 0.015	0.090	0.007 ± 0.012	0.063	Moderate but consistent contribution.
prod_mgm2	0.85	0.009 ± 0.017	0.099	0.019 ± 0.015	0.075	Moderate, stable effect across models.
genetic_condition	0.75	0.008 ± 0.018	0.064	0.008 ± 0.011	0.050	Small but consistent global genomic influence.
prod_wm1	0.55	0.012 ± 0.016	0.034	−0.000 ± 0.011	0.036	Weak, borderline signal.
prod_mgm12	0.70	0.003 ± 0.021	0.090	0.007 ± 0.013	0.059	Weak but reproducible across datasets.
prod_wm2	0.65	0.007 ± 0.015	0.035	−0.000 ± 0.011	0.042	Very small but consistent effect.
mut12_exon_class	0.55	−0.002 ± 0.016	0.051	0.023 ± 0.025	0.064	Small secondary exome-level influence.

Feature	Stability	CV Perm ± SD	CV Gini	Full Perm ± SD	Full Gini	Interpretation
prod_wmg	0.55	0.008 ± 0.014	0.019	−0.003 ± 0.012	0.051	Weak, borderline effect.
wolframin_class	0.35	0.007 ± 0.015	0.028	−0.000 ± 0.011	0.039	Minimal predictive value.
type_mut2_exon	0.40	0.003 ± 0.010	0.028	0.003 ± 0.010	0.018	Weak, likely noise.
type_mut1_exon	0.25	0.006 ± 0.012	0.022	−0.004 ± 0.013	0.018	Very weak and unstable.
prod_wm12	0.30	0.003 ± 0.016	0.031	−0.002 ± 0.012	0.041	Negligible signal.

Table S9. Anosmia — Relevant Predictors

In anosmia, mut1_protein_class and mut2_protein_class form the dominant predictors, with high stability and the largest importance values across all metrics. Mut2 displays the strongest overall effect, particularly in the full-model permutation and Gini scores, while mut1 shows a similarly substantial and consistent influence. This pattern indicates a clear protein-level signature driving the presence or absence of anosmia. A smaller but coherent secondary contribution comes from prod_mgm2, which remains consistently present across cross-validation and full-model metrics despite its low absolute magnitude. The interaction prod_wm2 shows weak yet reproducible signal and appears alongside wolframin_class, which also demonstrates a very small but stable effect across methods. Additional weak predictors include prod_mgm12, prod_wm1, and prod_wm12, each contributing small but detectable signals across multiple metrics. Genetic_Condition_class and prod_wmg appear with very low importance, indicating minimal discriminative value for this phenotype. Exome-level predictors (mut2_exom_class, mut12_exom_class, mut1_exom_class) remain weak and unstable, with low stability and inconsistent permutation effects, reflecting limited relevance once the dominant protein-class predictors are included. The global pattern highlights a phenotype driven primarily by protein-level effects, accompanied by small, dispersed contributions from specific interaction terms and negligible influence from exon-based or broader genetic-context variables. Anosmia is driven chiefly by protein-level effects, supported by small, consistent contributions from selected interaction terms and only minimal influence from the remaining genetic predictors.

Feature	Stability	CV Perm ± SD	CV Gini	Full Perm ± SD	Full Gini	Interpretation
mut1_protein_class	0.85	0.037 ± 0.033	0.203	0.059 ± 0.023	0.256	Strong genetic driver; stable across datasets.
mut2_protein_class	0.90	0.035 ± 0.026	0.204	0.088 ± 0.033	0.274	Strongest overall predictor; highly stable.
prod_mgm2	0.70	0.002 ± 0.018	0.099	0.002 ± 0.016	0.068	Moderate, consistent contribution.
prod_wm2	0.65	0.004 ± 0.013	0.035	0.010 ± 0.012	0.028	Weak but reproducible effect.
wolframin_class	0.60	0.005 ± 0.014	0.028	0.008 ± 0.011	0.028	Very small but stable signal.
type_mut2_exon	0.55	0.006 ± 0.009	0.028	−0.011 ± 0.018	0.043	Weak, inconsistent direction.
prod_mgm12	0.50	−0.005 ± 0.021	0.090	0.004 ± 0.011	0.049	Weak but present across models.
genetic_condition	0.45	−0.002 ± 0.016	0.064	0.000 ± 0.010	0.034	Very small effect.
prod_wm1	0.50	0.002 ± 0.016	0.034	0.008 ± 0.012	0.035	Weak but reproducible.
prod_mgm1	0.40	−0.006 ± 0.019	0.090	0.000 ± 0.014	0.055	Minimal and unstable.
prod_wm12	0.45	0.002 ± 0.014	0.031	0.005 ± 0.013	0.042	Very small effect.

Feature	Stability	CV Perm ± SD	CV Gini	Full Perm ± SD	Full Gini	Interpretation
prod_wmg	0.40	0.004 ± 0.014	0.019	0.000 ± 0.008	0.024	Negligible influence.
mut12_exon_class	0.30	−0.012 ± 0.017	0.051	0.016 ± 0.013	0.045	Very weak and unstable.
type_mut1_exon	0.20	−0.005 ± 0.013	0.022	−0.002 ± 0.019	0.021	Essentially noise.

Table S10. Tandem Gait Impairment — Relevant Predictors

In tandem gait impairment, mut2_protein_class remains the strongest and most stable predictor, carrying the highest importance across cross-validation and full-model metrics. This indicates that the protein effect on allele 2 is the primary determinant of impaired tandem gait. Mut1_protein_class contributes as a secondary but meaningful signal, particularly in the full permutation results, reinforcing a clear protein-level pattern behind the phenotype A consistent mid-level influence is observed for prod_mgm1, prod_mgm2, and prod_mgm12. These interaction terms, linking Genetic_Condition_Class with exon-related mutation structure, retain stable contributions despite their modest magnitude. Their presence suggests that zygosity–exon interactions subtly modulate tandem gait impairment once the main protein-level effects are established. Smaller yet reproducible effects are found in the interaction features prod_wm1 and prod_wm2, as well as in wolframin_class. Their limited but stable importance aligns with clinical expectations, as white-matter pathways and wolframin production type can influence cerebellar and brainstem circuits relevant to balance and coordination. The general zygosity variable Genetic_Condition_class shows very low discriminative power, and prod_wmg behaves similarly, indicating that broader genetic-context labels add little once protein-class and interaction features are included. Exon-level predictors (mut1_exom_class, mut2_exom_class, mut12_exom_class) remain weak and unstable, reflecting limited relevance after the protein-level consequences are accounted for. Tandem gait impairment is characterized by a strong protein-level signature, dominated by the functional impact of mutations on allele 2 and supported by a notable contribution from allele 1. Interaction terms involving zygosity and exon-level mutation classes provide a secondary layer of influence, while white-matter–related interactions and wolframin production class add small, reproducible effects. Exon-based predictors and broad genetic-context variables offer little explanatory value once protein-level and interaction features are considered.

Feature	Stability	CV Perm ± SD	CV Gini	Full Perm ± SD	Full Gini	Interpretation
mut2_protein_class	1.00	0.055 ± 0.027	0.204	0.103 ± 0.034	0.281	Dominant and most stable predictor; strong genetic driver.
mut1_protein_class	0.75	0.003 ± 0.028	0.203	0.047 ± 0.017	0.189	Secondary protein-level effect; strong in full model.
prod_mgm1	0.85	0.017 ± 0.019	0.090	0.010 ± 0.008	0.052	Consistent moderate predictor.
prod_mgm2	0.80	0.009 ± 0.023	0.099	0.014 ± 0.010	0.070	Stable functional influence across datasets.
prod_mgm12	0.75	0.007 ± 0.023	0.090	0.007 ± 0.008	0.055	Weak but reliable secondary contributor.
prod_wm1	0.65	0.009 ± 0.014	0.034	0.004 ± 0.009	0.046	Weak but consistent white-matter influence.
prod_wm2	0.60	0.006 ± 0.013	0.035	0.006 ± 0.009	0.053	Small but meaningful effect.
genetic_condition	0.55	0.001 ± 0.020	0.064	0.009 ± 0.008	0.038	Weak, general genetic context effect.
wolframin_class	0.50	0.009 ± 0.013	0.028	0.005 ± 0.008	0.039	Very small influence.

Feature	Stability	CV Perm ± SD	CV Gini	Full Perm ± SD	Full Gini	Interpretation
type_mut1_exon	0.40	0.009 ± 0.008	0.022	0.003 ± 0.007	0.025	Weak secondary genetic signal.
type_mut2_exon	0.35	0.008 ± 0.011	0.028	0.003 ± 0.013	0.037	Very small effect.
prod_wm12	0.40	0.001 ± 0.013	0.031	0.005 ± 0.009	0.047	Minimal but non-random.
mut12_exon_class	0.30	0.001 ± 0.013	0.051	0.006 ± 0.010	0.046	Very weak and unstable.
prod_wmg	0.25	0.007 ± 0.012	0.019	0.002 ± 0.007	0.023	Negligible influence.

Table S11. Dysarthria— Relevant Predictors

In dysarthria, mut1_protein_class and mut2_protein_class form the leading predictors, with the highest importance and strongest stability across both cross-validation and full-model analyses. The two alleles show nearly identical influence, indicating that the protein-level functional consequences of both mutations are central to the presence and severity of dysarthria. These results point to a clear protein-level signature, where loss or dysfunction of wolframin from either allele substantially increases the likelihood of speech articulation impairment. A substantial secondary contribution arises from mut12_exom_class, which appears consistently among the top-ranked features in both settings. This pattern suggests that whether both mutations fall within the same exon or in different exons adds relevant structural information once the protein-level effects are accounted for, possibly reflecting shared mechanistic pathways linked to exon-specific vulnerability. Stable mid-range signals are observed in the interaction features prod_mgm1, prod_mgm12, and prod_mgm2, all linking Genetic_Condition_Class with exon-level mutation classes. These interactions contribute modest but reliable effects, indicating that zygosity–exon coupling modulates dysarthria risk in a reproducible manner. Their presence suggests nuanced influences of gene dosage and mutation positioning on motor-speech circuitry. Weaker but reproducible signals emerge from the white-matter interaction terms prod_wm1, prod_wm2, and prod_wm12, alongside small contributions from wolframin_class. Although limited in magnitude, these effects align with the involvement of white-matter pathways and wolframin production type in the neural systems supporting speech motor control. Broad genetic-context variables such as Genetic_Condition_class and prod_wmg show very low importance values, indicating that broad genetic categories add little once detailed mutation classes and protein-level effects are included. Single-exon predictors (mut1_exom_class, mut2_exom_class) also remain weak and unstable, reflecting minimal discriminative value beyond the richer interaction and protein-level features. Dysarthria is dominated by strong, symmetric protein-level effects from both alleles, with a notable contribution from mut12_exom_class and consistent modulation from zygosity–exon interaction terms. White-matter interactions and wolframin production class provide smaller but reproducible influences, while broad genetic-context and isolated exon-level variables contribute minimally after the detailed mutation classes are incorporated.

Feature	Stability	CV Perm ± SD	CV Gini	Full Perm ± SD	Full Gini	Interpretation
mut1_protein_class	0.95	0.074 ± 0.037	0.203	0.117 ± 0.047	0.266	Strongest and most stable predictor; protein-impact severity is central to dysarthria.
mut2_protein_class	0.95	0.041 ± 0.034	0.204	0.125 ± 0.039	0.245	Co-dominant driver; mutation 2 protein disruption closely tied to phenotype.
mut12_exon_class	0.80	0.047 ± 0.018	0.051	0.074 ± 0.027	0.096	Stable combined exome effect; meaningful but secondary to protein-level classes.
prod_mgm12	0.75	0.011 ± 0.022	0.090	0.021 ± 0.023	0.089	Moderately important MRI-derived brainstem/cerebellar composite feature.
prod_mgm2	0.70	0.001 ± 0.019	0.099	0.019 ± 0.020	0.060	Consistent weak-to-moderate contribution; likely midbrain/pons volume relevance.

Feature	Stability	CV Perm ± SD	CV Gini	Full Perm ± SD	Full Gini	Interpretation
prod_mgm1	0.65	0.015 ± 0.020	0.090	0.008 ± 0.014	0.043	Small but reproducible effect; part of brainstem structural signal.
prod_wm12	0.55	0.002 ± 0.020	0.031	0.013 ± 0.021	0.033	Mild, consistent white-matter microstructural influence.
prod_wm2	0.50	0.007 ± 0.020	0.035	0.008 ± 0.016	0.025	Weak but non-random WM marker.
prod_wm1	0.45	0.012 ± 0.022	0.034	0.003 ± 0.013	0.024	Small signal but directionally stable.
genetic_condition	0.40	0.002 ± 0.018	0.064	0.003 ± 0.011	0.035	Weak global genetic-context label; overshadowed by specific protein classes.
type_mut1_exon	0.35	0.012 ± 0.016	0.022	0.006 ± 0.016	0.023	Minor secondary exome effect; redundant with protein-level classification.
wolframin_class	0.30	0.005 ± 0.019	0.028	0.003 ± 0.013	0.020	Broad genetic-context labels contribute minimal information beyond what is already captured by the detailed mutation-class features.
prod_wmg	0.25	0.004 ± 0.016	0.019	0.010 ± 0.014	0.019	Very weak global WM measure; minimal discriminatory value.
type_mut2_exon	0.20	0.002 ± 0.012	0.028	0.003 ± 0.011	0.022	Low contribution; largely redundant with mut2_protein_class.

Table S12. Adiadochokinesia – Relevant Predictors

In adiadochokinesia, mut1_protein_class and mut2_protein_class form the dominant predictors, with the highest importance and strongest stability across cross-validation and full-model metrics. Their nearly identical influence indicates that the phenotype is strongly shaped by the protein-level functional impact of both alleles, fitting the pattern observed in other cerebellar-related motor findings in Wolfram syndrome. These two variables create a clear protein-level signature corresponding to the loss or dysfunction of wolframin in circuits governing rapid alternating movements. A secondary contribution arises from mut12_exom_class, which consistently appears among the higher-ranked features in both analyses. This suggests that whether both mutations lie within the same exon or in different exons provides additional structural information once the protein effects are established, possibly capturing exon-specific vulnerability relevant to cerebellar timing and coordination. Stable mid-range influences appear in the interaction features prod_mgm1, prod_mgm12, and prod_mgm2, which link Genetic_Condition_Class × exon-level mutation classes. These interaction terms contribute modest but reliable effects, indicating that zygosity–exon structure interactions modulate the phenotype beyond the primary protein-class determinants. Their consistency aligns with the role of gene dosage and mutation positioning in circuits responsible for fine motor sequencing. Small but reproducible signals are found in the white-matter interaction features prod_wm1, prod_wm2, and prod_wm12, alongside limited contributions from wolframin_class. Although subtle, these effects match the involvement of white-matter pathways and wolframin production class in the networks responsible for smooth, alternating motor output. Broad genetic-context variables such as Genetic_Condition_class and prod_wmg show very low discriminative value, indicating that broad genetic categories add little captured by detailed mutation classes. Exon-specific predictors (mut1_exom_class and mut2_exom_class) also remain weak and unstable, contributing minimal information once the core protein-level and interaction effects are considered. Adiadochokinesia is driven primarily by strong, symmetric protein-level effects from both alleles, with additional information provided by mut12_exom_class and consistent contributions from zygosity–exon interaction terms. White-matter interactions and wolframin production class exert small but reproducible influences, while broad genetic-context variables and isolated exon-level features contribute little after accounting for the detailed mutation classes.

**Feature**	**Stability**	**CV Perm ± SD**	**CV Gini**	**Full Perm ± SD**	**Full Gini**	**Interpretation**
mut1_protein_class	0.95	0.076 ± 0.031	0.203	0.066 ± 0.030	0.198	Strongest, highly stable predictor; major protein-level determinant.
mut2_protein_class	0.95	0.060 ± 0.027	0.204	0.057 ± 0.024	0.202	Co-dominant driver; robust protein-pathogenicity effect.
prod_mgm1	0.80	0.012 ± 0.013	0.090	0.008 ± 0.015	0.095	Most consistent MRI predictor; brainstem–cerebellar involvement.
mut12_exon_class	0.70	0.023 ± 0.016	0.051	0.015 ± 0.013	0.048	Moderate combined-exome influence; secondary to protein-level classes.
prod_mgm12	0.65	0.005 ± 0.016	0.090	0.003 ± 0.016	0.085	Weak but reproducible brainstem–cerebellar composite signal.
prod_wm1	0.60	0.012 ± 0.017	0.034	0.006 ± 0.015	0.035	Small but stable white-matter contribution.

Feature	Stability	CV Perm ± SD	CV Gini	Full Perm ± SD	Full Gini	Interpretation
prod_wm2	0.60	0.009 ± 0.015	0.035	0.006 ± 0.015	0.038	Weak but consistent white-matter effect.
prod_mgm2	0.55	0.000 ± 0.013	0.099	-0.005 ± 0.015	0.104	Very small and unstable MRI signal; may reflect noise or subtle ROI changes.
prod_wm12	0.50	0.005 ± 0.016	0.031	0.005 ± 0.015	0.033	Minimal but non-random white-matter component.
wolframin_class	0.40	0.006 ± 0.015	0.028	0.005 ± 0.014	0.027	Weak broad mutation category; limited added value.
genetic_condition	0.35	0.000 ± 0.013	0.064	-0.002 ± 0.016	0.066	Minimal effect; overshadowed by specific mutation classes.
type_mut1_exon	0.35	0.008 ± 0.012	0.022	0.006 ± 0.015	0.023	Very small secondary exome signal.
prod_wmg	0.30	0.005 ± 0.014	0.019	0.004 ± 0.011	0.025	Negligible global white-matter effect.
type_mut2_exon	0.25	0.001 ± 0.010	0.028	0.001 ± 0.014	0.020	Weakest genetic signal; largely redundant.

Figure S1. Cross-validated ROC curves for each neurological disorder. Each panel shows the mean receiver operating characteristic (ROC) curve obtained using repeated stratified cross-validation. For each symptom, models were trained and evaluated using 5-fold cross-validation with multiple repetitions (20), and the ROC curves from all folds were aggregated. The solid line represents the mean ROC across all repetitions, and the shaded area corresponds to the ±1 standard deviation interval, reflecting fold-to-fold variability. The mean AUC and its standard deviation quantify the robustness and stability of model performance for each neurological outcome. The ROC curves indicate moderate discriminative performance across the neurological outcomes, with substantial variability between symptoms. For most disorders, the mean ROC curve lies above the diagonal reference line, demonstrating that the models extract some predictive signal from the available clinical and genetic variables. Symptoms such as ataxia, dysmetria, and gait instability exhibit relatively higher AUC values and narrower variability bands, suggesting more reliable discrimination and greater model stability across the 100 resamples (20 repetitions × 5 folds). In contrast, disorders such as dysphagia, sialorrhea, and anosmia show more modest AUCs and wider confidence bands, indicating weaker predictive signal and higher sensitivity to fold partitioning. The width of the shaded regions reflects the degree of uncertainty introduced by small sample sizes and imbalanced class distributions. Although the models retain some discriminative capacity, the fold-to-fold variability underscores the challenge of predicting certain neurological manifestations in a rare disease context. The cross-validated ROC curves highlight that the models perform reasonably well for some neurological features while offering limited predictive value for others. These findings should

be interpreted within the multisystemic and heterogeneous nature of Wolfram syndrome and the constraints inherent to small-cohort machine-learning analyses.

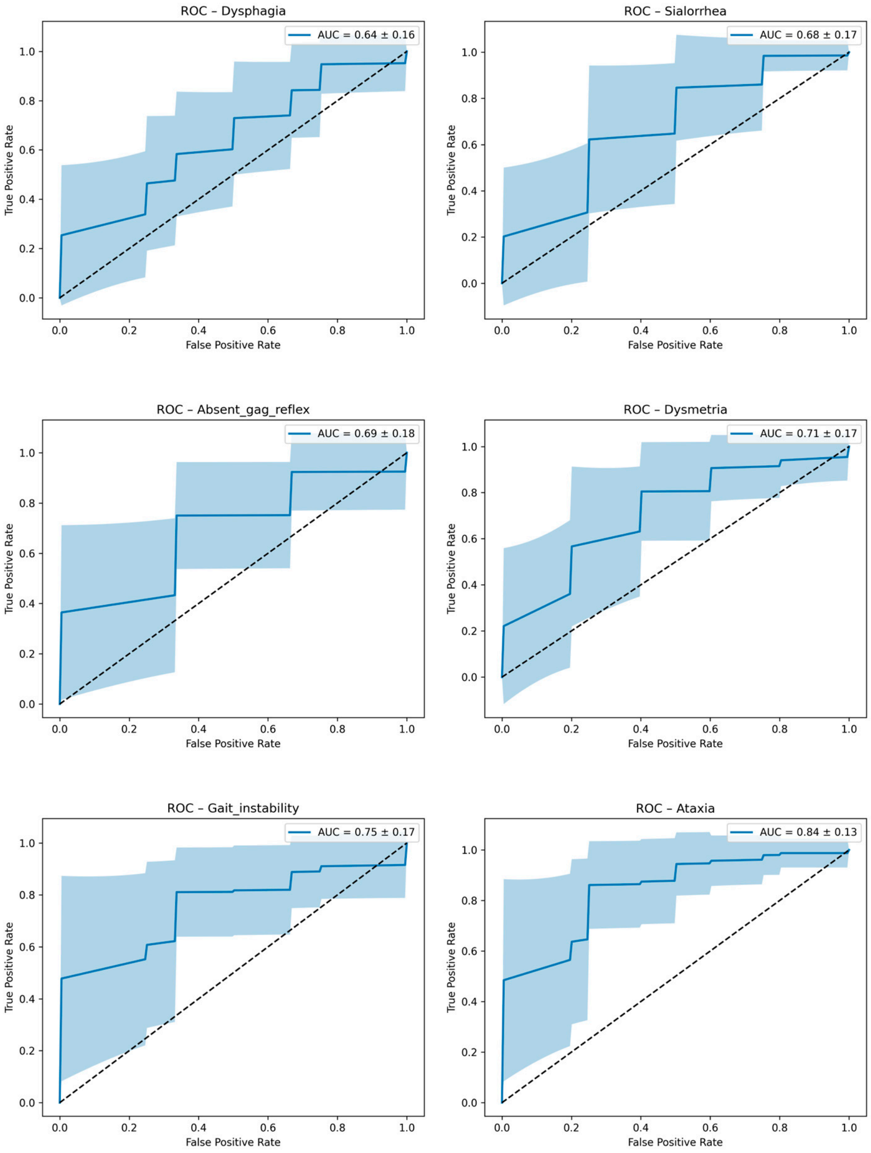

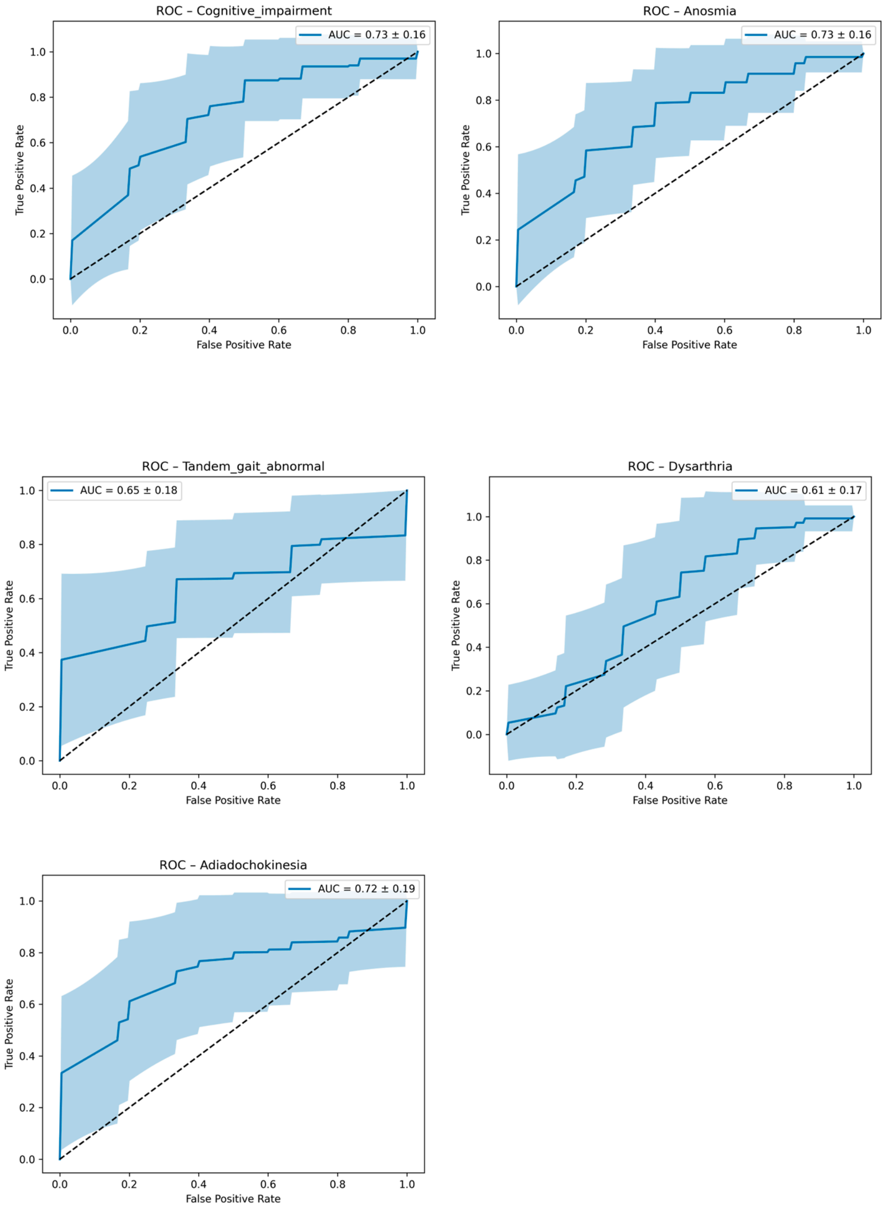
ROC – Cognitive_impairment
AUC = 0.73 ± 0.16
True Positive Rate
False Positive Rate
ROC – Anosmia
AUC = 0.73 ± 0.16
True Positive Rate
False Positive Rate
ROC – Tandem_gait_abnormal
AUC = 0.65 ± 0.18
True Positive Rate
False Positive Rate
ROC – Dysarthria
AUC = 0.61 ± 0.17
True Positive Rate
False Positive Rate
ROC – Adiadochokinesia
AUC = 0.72 ± 0.19
True Positive Rate
False Positive Rate
